# EpInflammAge: Epigenetic-Inflammatory Clock for Disease-Associated Biological Aging Based on Deep Learning

**DOI:** 10.3390/ijms26136284

**Published:** 2025-06-29

**Authors:** Alena Kalyakulina, Igor Yusipov, Arseniy Trukhanov, Claudio Franceschi, Alexey Moskalev, Mikhail Ivanchenko

**Affiliations:** 1 Artificial Intelligence Research Center, Institute of Information Technologies, Mathematics and Mechanics, Lobachevsky State University, Nizhny Novgorod 603022, Russia; 2 Institute of Biogerontology, Lobachevsky State University, Nizhny Novgorod 603022, Russia; 3 Mriya Life Institute, National Academy of Active Longevity, Moscow 124489, Russia

**Keywords:** aging, biological clock, inflammaging, DNA methylation, inflammatory profile, deep neural network, explainable artificial intelligence

## Abstract

We present EpInflammAge, an explainable deep learning tool that integrates epigenetic and inflammatory markers to create a highly accurate, disease-sensitive biological age predictor. This novel approach bridges two key hallmarks of aging—epigenetic alterations and immunosenescence. First, epigenetic and inflammatory data from the same participants was used for AI models predicting levels of 24 cytokines from blood DNA methylation. Second, open-source epigenetic data (25 thousand samples) was used for generating synthetic inflammatory biomarkers and training an age estimation model. Using state-of-the-art deep neural networks optimized for tabular data analysis, EpInflammAge achieves competitive performance metrics against 34 epigenetic clock models, including an overall mean absolute error of 7 years and a Pearson correlation coefficient of 0.85 in healthy controls, while demonstrating robust sensitivity across multiple disease categories. Explainable AI revealed the contribution of each feature to the age prediction. The sensitivity to multiple diseases due to combining inflammatory and epigenetic profiles is promising for both research and clinical applications. EpInflammAge is released as an easy-to-use web tool that generates the age estimates and levels of inflammatory parameters for methylation data, with the detailed report on the contribution of input variables to the model output for each sample.

## 1. Introduction

Aging is characterized by progressive deterioration of cellular, tissue, physiological, and cognitive functions, manifesting through multiple interconnected biological mechanisms that collectively impact organismal health and longevity [1,2]. It is followed by an increased susceptibility to age-associated pathologies, including cancer, metabolic disorders, cardiovascular diseases, and neurodegenerative conditions [3,4,5,6]. According to a broadly accepted classification, there are nine basic hallmarks of aging: genomic instability, telomere attrition, epigenetic alterations, loss of proteostasis, deregulated nutrient sensing, mitochondrial dysfunction, cellular senescence, stem cell exhaustion, and altered intercellular communication [4]. While their interconnectedness is beyond doubt, disentangling it still remains a challenge. Here, we aim to advance bridging age-related epigenetic modifications and immunosenescence.

One of the most frequently addressed epigenetic mechanisms of aging is DNA methylation, which can lead to genomic instability, disrupting gene regulation and cellular processes, and contribute to the development of age-related diseases [1,7,8]. Age-related changes are also linked to immune system remodeling [9,10,11], the development of persistent inflammation, and the associated imbalance between pro- and anti-inflammatory mechanisms [12,13]. This underlies the concept of inflammaging, a major paradigm of immunosenescence [14]. Inflammaging is defined by continuous remodeling of the immune system, which is characterized by a gradual decline in immune function with age. This process is associated with chronic low-grade inflammation, which is implicated in the aging process and the development of age-related diseases [15,16]. Age-related epigenetic remodeling is closely linked to inflammaging: epigenetic alterations contribute to chronic inflammation and impaired anti-inflammatory processes [17,18,19,20], and inflammation promotes heterochromatin loss and age-related hypomethylation [21,22,23].

Aging clock models are widely used to estimate an individual’s biological age, which reflects the current health status and the risk of developing age-related diseases. Among them, epigenetic clocks receive much attention due to the good standardization and accumulating availability of DNA methylation data, and model development has surpassed several generations by now [24,25,26,27,28,29,30]. Such models have also been studied in the context of sensitivity to different disease groups (cardiovascular, neurodegenerative, oncological, endocrine, psychiatric, viral), namely, by the ability to detect accelerated aging in patients [31,32,33]. Fewer tools exist for the assessment of inflammaging, and single markers may not be sufficient to capture the complexity of this process. Currently developed models utilize different sets of markers (various plasma proteins, including cytokines and chemokines) to estimate age and are sensitive to certain age-related pathologies, including cardiac, renal, and neurodegenerative diseases [34,35,36,37,38,39]. The acceleration of epigenetic and inflammatory aging (defined as a discrepancy between biological age and chronological age) is influenced by a multitude of factors, including, but not limited to, diseases and pathological conditions, sex, race/ethnicity, environment, socioeconomic status, stress, and others [7,31,40,41,42].

In order to incorporate aging clocks in clinical practice, it is necessary that they demonstrate sensitivity to a wide range of patient pathologies, with these sensitivities being influenced by numerous factors. In the context of epigenetic models, the sensitivity to diseases may depend on the selected set of features (CpG sites), the tissue (different pathologies affect changes in different tissues in different ways), the training data, and numerous other parameters [32,43,44]. Immunological clocks are capable of detecting the presence of inflammation associated with disease and age (inflammaging) [34,35,39]. To study and assess the aging of more isolated body systems (e.g., the brain), completely different models may be required. These models would be based on analyses of cerebrospinal fluid, brain electroencephalography signals, or magnetic resonance imaging [45,46]. The integration of diverse tissue types and medical tests within the aging clock model has the potential to enhance the system’s reliability and sensitivity to various pathologies [24,47].

The convergence of two hallmarks of aging, epigenetic modifications and immunosenescence, can be investigated by a simultaneous analysis of the two profiles: epigenetic and immunological/inflammatory. However, the limited prevalence of immunological data represents a significant challenge. A possible solution could make use of the recently proposed methodology of estimating the levels of individual blood plasma proteins from DNA methylation data [48,49], with a further objective of mortality or age prediction [50,51,52]. This approach can also underpin the construction of aging clock models and uncover associations with various pathologies. Since many blood plasma cytokines and chemokines possess pro- and anti-inflammatory properties [53,54,55], it will be feasible to evaluate inflammaging in the blood through the use of epigenetics.

In this paper, we present an AI-based approach that infers the inflammatory profile (cytokines and chemokines levels) from DNA methylation data, and makes use of it for the epigenetic-inflammatory clock model (EpInflammAge). Epigenetic data from more than 25,000 open-access samples were used to develop the age predictor model. We performed an extensive comparison of EpInflammAge to 34 existing epigenetic age models both in terms of age prediction quality metrics (mean absolute error, correlation coefficient) for healthy samples and sensitivity to different diseases (covering 13 chapters of ICD-11). The impact of individual inflammatory parameters on age prediction was analyzed using explainable AI approaches.

For ease of application, the proposed model is published together with a user-friendly web interface, which only requires uploading a table with methylation data and age. The interface is able to handle the missing values in the data by providing options for imputation, which—together with the estimation of inflammatory parameters—provides a significant added value to epigenetic age estimation. The web interface also produces plots of predicted age as a function of chronological age and the distribution of age acceleration. Detailed explanations of the effect of each feature on the age estimate and the significance of each inflammatory marker in a particular sample compared with the overall distribution of similarly aged individuals is also implemented. The web interface is openly available from the HuggingFace platform [56].

## 2. Results

### 2.1. Study Design and Model Development Pipeline

**Figure 1:**
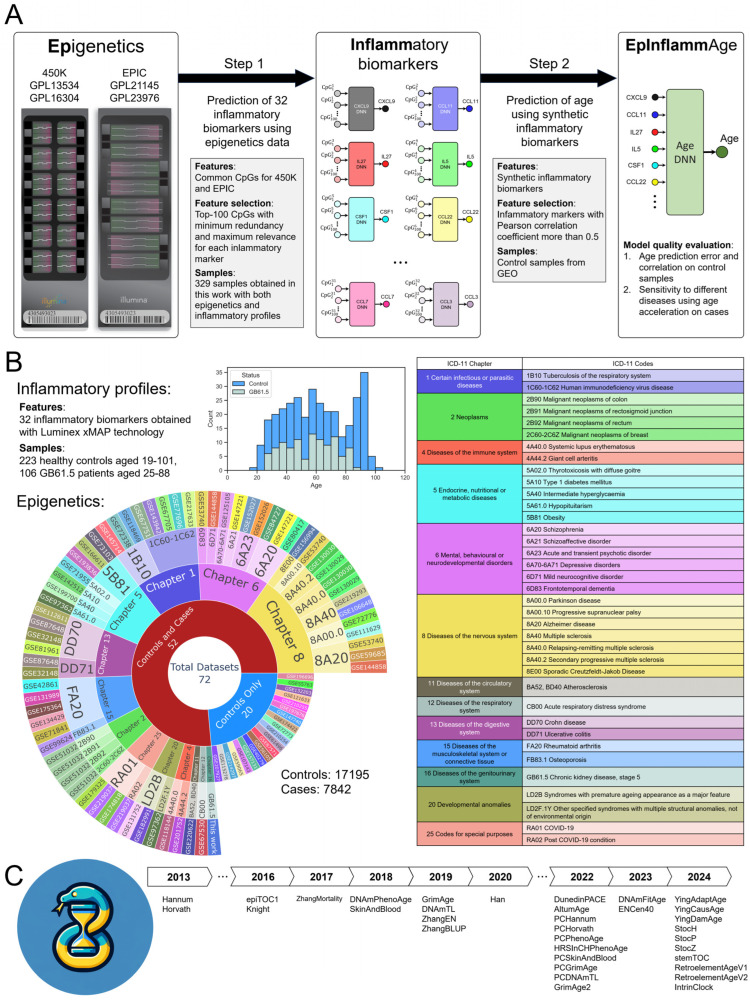
Study design. (**A**) Main steps in the development of the epigenetic-inflammatory (EpInflammAge) clock model. First, inflammatory markers were estimated from simultaneous epigenetic profile data. As a result, we obtained 32 neural network models (one for each inflammatory marker) predicting inflammatory marker levels. Second, the age estimation model based on synthetic inflammatory parameters derived from the open source methylation data was developed and evaluated against controls and cases groups. (**B**) Description of data used in this study. The histogram represents the age distribution of the participants whose data were used at the first step. The sunburst diagram characterizes the open source epigenetic data used at the second step: the number of datasets, the number of datasets with controls and cases; ICD-11 chapters and codes for diseases in the studied case groups. The outer level of the diagram gives datasets codes in the NCBI GEO repository. The table on the right accounts for the chapters and particular ICD-11 codes; the samples with these codes were used for the disease-sensitivity test of the resulting clock model. (**C**) Timeline of development of epigenetic clock models (calculated via pyaging) assessed in our study.

EpInflammAge was constructed in two steps. First, we established the correspondence between epigenetic and inflammatory parameters and built a set of AI models that predict the levels of plasma cytokines and chemokines based on DNA methylation data. Second, we produced a two-level biological clock model that employs the AI-predicted levels of inflammatory parameters as an input for the AI-based age estimate (Figure 1A,B).

The first step utilized simultaneous epigenetic and inflammatory profile data. We make use of the blood sample analysis results for 329 residents of the Russian Federation, aged 19 to 101 years, and recruited in 2019–2023 [35,38,57]. Participants included a group of healthy controls (223 participants, 19 to 101 years old) and a group of chronic renal disease patients (106 participants, 25 to 88 years old), the latter broadening the scale of biomarker values, given a certain difference in epigenetic and inflammatory profiles between patients and healthy controls [38]. The healthy control group included the representatives from the general population without chronic diseases in the acute phase, cancer, acute respiratory infections, and pregnancy; the chronic renal disease group included patients with stage 5 disease regularly undergoing dialysis. The set of analyzed CpG sites corresponds to the probes common to Illumina 450k and EPIC standards in order to ensure a broader applicability of EpInflammAge, and inflammatory profiles are given by levels of 32 cytokines and chemokines.

To estimate inflammatory parameters based on epigenetic profiles, we first used the mRMR approach (minimum Redundancy–Maximum Relevance, which chooses subset of features mostly associated with the target variable) for feature selection and reducing redundancy [58]. As a result, we obtained sets of 100 CpG sites for each of the 32 inflammatory markers, and found them to have a fairly low overlap (altogether, 2936 different CpG sites were selected to construct predictors for 32 inflammatory markers cf. Supplementary Table S1). Next, we constructed 32 separate deep neural networks, one for each inflammatory parameter, with 32 different sets of 100 CpG sites serving as inputs. We used state-of-the art deep neural network architectures for tabular data with training, validation and test subset divisions; cross-validation and hyperparametric search were used to select optimal hyperparameter sets. The procedure is illustrated in Figure 1A, Step 1.

The second step addressed age prediction from synthetic inflammatory markers (Figure 1A, Step 2). At this stage, we made use of public blood DNA methylation datasets from the NCBI GEO repository [59]. We limited our search to Illumina 450k and EPIC standard methylation datasets that have age and sex annotation. Altogether, the data from 17,195 healthy participants from 72 datasets with the overall age range starting from a few months to 101 years were used to build the clock. We performed feature selection, retaining only inflammatory parameters with the correlation coefficient between real and predicted values greater than 0.5 (see Section 2.2 for details). Synthetic values of inflammatory parameter levels were calculated by means of the models developed in the first step. The same model training process was used at this step.

Further on, the data from patients with various diseases were used to analyze the sensitivity of the clock model to ICD-11 (see Section 2.4 for details). We also compared the performance of EpInflammAge with multiple existing epigenetic clocks (Figure 1C).

### 2.2. Predicting Inflammatory Biomarkers from Epigenetic Data

**Figure 2:**
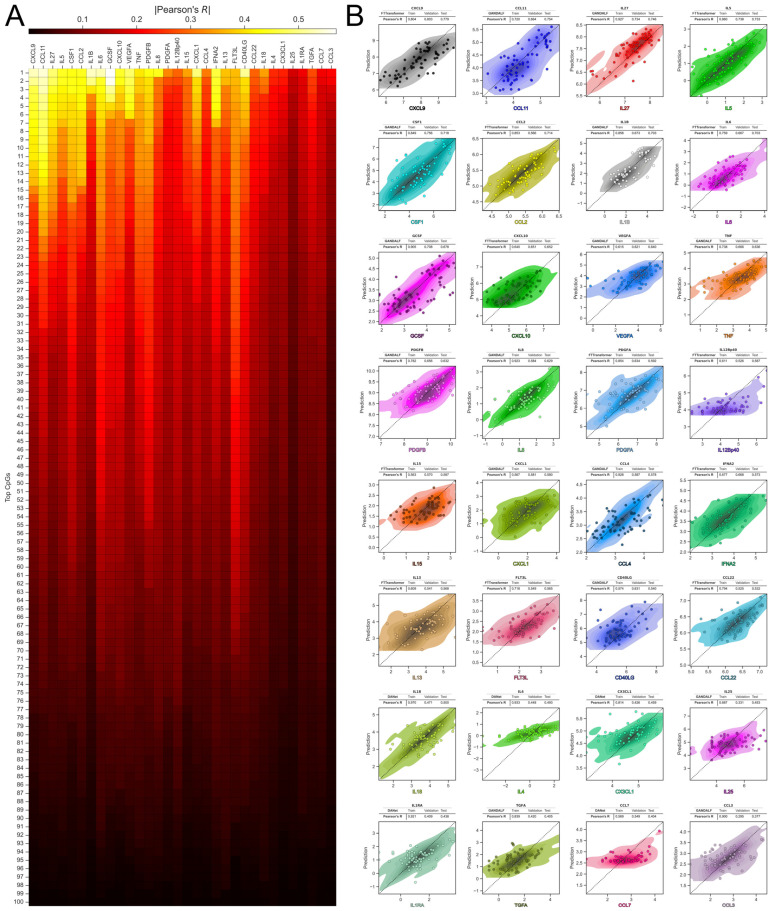
Results of the evaluation of inflammatory parameters based on DNA methylation data. (**A**) Correlation map representing the relationship between inflammatory parameters and the top 100 CpG sites for each. The absolute value of the Pearson correlation coefficient is shown in color. (**B**) Results of neural network models for all inflammatory markers. For each inflammatory marker, the relationship between real and predicted levels is presented (logarithmic scale), KDE corresponds to training and validation data, scatter corresponds to test data. The table for each inflammatory marker shows the Pearson correlation coefficient for training, validation, and test data separately. The architecture for the best final model is also presented.

We start with building deep neural network models that estimate the levels of inflammatory markers taking epigenetic data as the input (one neural network–one inflammatory marker). First, for each inflammatory marker, we selected the top 100 CpG sites according to the mRMR approach (cf. listed results in Supplementary Table S2) and investigated corresponding Pearson correlation coefficients (Pearson’s R) between levels of inflammatory markers and their CpGs. The best absolute values of the correlation coefficient were observed for the top CpG sites used to predict levels of CXCL9, CCL11, IL1B, IL6, and GCSF (heatmap at Figure 2A).

Next, we trained individual neural networks for different inflammatory markers based on specific top 100 CpG sites (see Methods for details on CpG site selection and the number of them). In Figure 2B, we illustrate the relationships between the real and predicted values (in logarithmic scale) and annotate the main characteristics of each model (best model architecture, MAE—mean absolute error, and correlation coefficient for all subsets). The results indicate a varying quality of model performance in individual inflammatory marker predictions. In the test data, the best prediction result was achieved for CCL9, with a correlation coefficient of 0.78. It is noteworthy that the lowest result was for CCL3, with a correlation coefficient of 0.37 (which is even higher than the acceptable minimum threshold for surrogate biomarkers in GrimAge [27], and the average correlation between measured inflammatory biomarkers and EpiScores in [50]). To ensure the reliability of the features for further age prediction, we set the correlation coefficient threshold at 0.5. A total of 24 out of 32 inflammatory markers analyzed passed the threshold. ElasticNet, one of the most well-known approaches in clock model building, was utilized as a baseline, but it did not achieve the top ranking for any inflammatory marker, as would be expected. This traditional linear model is quite dated, and in this instance, it was outperformed by modern deep neural networks, which are capable of finding more complex dependencies in the data. The detailed results for all inflammatory markers and all model architectures tested are in Supplementary Table S3 and Supplementary Figure S1.

**Figure 3:**
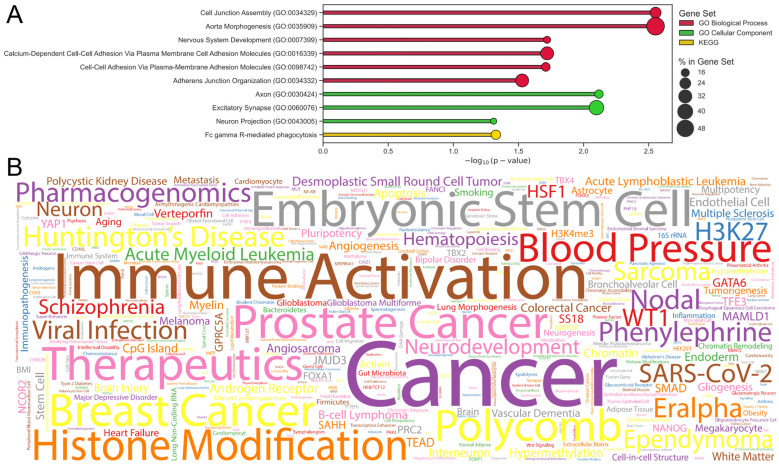
GSEA results for the list of genes involved in the development of inflammatory parameter estimation models. (**A**) GSEA results for GO Biological Process (red), GO Cellular Component (green), and KEGG (yellow) libraries. The plot shows the FDR-adjusted *p*-value on a logarithmic scale. The size of the circle for each term shows the percentage of coverage of the gene list corresponding to the term. (**B**) Word cloud demonstrating the keywords of the papers corresponding to the most significant terms. The size of words corresponds to the frequency of occurrence: the larger the word, the more papers corresponding to the terms have it as a keyword.

We identified the genes for the CpG sites participating in inflammatory marker prediction models (Supplementary Table S4) and performed Gene-Set Enrichment Analysis (GSEA). For this purpose, we used the Enrichr tool, which aggregates a large number of various libraries with lists of genes corresponding to different structures and processes in the human body [60,61,62]. The resulting list of all significant terms that were found in the libraries is given in Supplementary Table S5. The most common and comprehensive term libraries are Gene Ontology (GO) [63] and KEGG [64], so we considered the significant terms (with FDR-adjusted *p*-value < 0.05) from these libraries separately (Figure 3A). In the GO Biological Process library, the terms related to junction organization and assembly (cell junction assembly and adherence junction organization), cell adhesion (calcium-dependent cell-cell adhesion via plasma membrane cell adhesion molecules and cell-cell adhesion via plasma-membrane cell adhesion molecules), as well as aortic morphogenesis and nervous system development were found to be significant. Axon, excitatory synapse and neuron projection terms found in GO Cellular Component are also relevant. Many terms related to cellular compounds can be associated with aging as well as with the activation and functioning of the immune system during the onset and spread of various diseases and during wound healing [65,66,67,68,69]. The functioning of the nervous system declines with age, including degeneration of axons and synapses, which is associated, among other things, with the development of age-associated neurodegenerative diseases [70,71]. The only significant term in the KEGG library concerned phagocytosis, a process that is actively involved in host-defense mechanisms, through which cells recognize, engulf, and digest extrinsic particles (bacteria, apoptotic cells, cellular debris, and others) [72]. Interestingly, phagocytosis being closely related to the functioning of the immune system and maintenance of homeostasis is subject to changes during aging, and phagocyte dysfunction with age may lead to the development of age-associated diseases [73]. Some noteworthy terms from the Enrichr-based list include: epigenetic rejuvenation of mesenchymal stromal cells derived from induced pluripotent stem cells [74]; NF90 target proteins IRF3 and IRF9, crucial regulators of the interferon pathway involved in immune response [75]; pioneer transcription factor ASCL1 regulating neurogenesis and chromatin remodeling [76], and age-associated changes in lncRNA expression and immune system processes [77]. We additionally visualize the results using the word cloud, the word size corresponding to the frequency of the keywords from the papers that investigate the found terms (Figure 3B). Immune activation, cancer (and different individual types of cancer), histone modification, blood pressure, and embryonic stem cells proved to be the most common. It should be pointed out that both aging-related tags and those related to immune function and disease emerged in this word cloud, which suggests potential interconnectedness in the context of epigenetic and inflammatory aging.

### 2.3. EpInflammAge: Epigenetic-Inflammatory Clock Model

**Figure 4:**
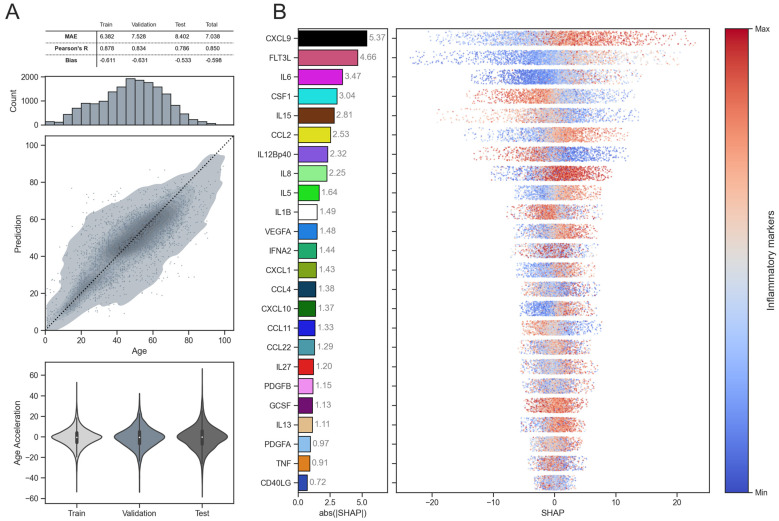
Results of EpInflammAge epigenetic-inflammatory clock model. (**A**) (1st row) Main metrics (MAE, Pearson correlation coefficient, bias) total, and separated for training, validation, and test subsets. (2nd row) Histogram of the age distribution of samples used for model training, validation, and testing. (3rd row) Dependence of the predicted age on the real age for the training and validation subsets (gray KDE), and for the test subset (gray scatter). The black dashed line corresponds to the bisector. (4th row) Violin plots of age acceleration value distributions for training, validation, and test data. (**B**) Applying XAI to the EpInflammAge model. (left) Bar plot illustrating the absolute SHAP values for all inflammatory parameters in descending order. (right) Illustration of associations between inflammatory markers values and SHAP values. Higher values of each inflammatory marker are in red, lower values in blue.

Figure 4 summarizes the main characteristics of the EpInflammAge model, which predicts age from epigenetic data with an intermediate embedding in the form of synthetic inflammatory parameters, thereby reflecting both epigenetic and inflammatory profiles. The baseline ElasticNet linear model and several deep neural network architectures for tabular data were used to build a biological age estimation model (detailed results for all architectures are in Supplementary Table S3 and Supplementary Figure S1). The resulting model is based on the DANet (Deep Abstract Network) deep neural network architecture and demonstrates a good performance, with a MAE of 7 years, and a correlation between the real and predicted age of 0.85 (the detailed results for training, validation and test models are in Figure 4A, all samples used in model construction are listed in Supplementary Table S6). It is worth noting that the model demonstrates a low bias, partly due to the fact that a modern model, specialized for tabular data, is used (linear models, widely used for epigenetic clocks, yield considerable systematic errors in predicted age [41]). The model was built using epigenetic profiles from more than 17,000 samples without diseases and covering a very wide age range (0–101 years). The use of such a large amount of training data of different standards was intended to increase the reliability of the results, even out batch effects from the methylation data collection and processing conditions, and help the model become more familiar with the wide variability of epigenetic data. Utilizing only healthy controls during model training prevents data leakage when evaluating the model’s performance on disease samples. The violin plots for age acceleration values for the training, validation, and test data are similar and sufficiently wide, and the quality metrics do not differ significantly. This suggests that the model is not overfitted on the training data (hereinafter, age acceleration refers to the difference between biological and chronological age).

Figure 4B shows the results of applying explainable AI approaches to the EpInflammAge model. The features (synthetic inflammatory parameters) are ranked according to their influence on the model’s final decisions as a whole, measured by SHAP (Shapley Additive Explanations, a game theory approach to measure influence of each variable of the model prediction) values (so-called global explainability). The largest contribution comes from CXCL9, which agrees with the previous results [34,35]. Higher values of CXCL9 correspond to a positive contribution to age estimation, while lower values correspond to a negative contribution. Similar associations of inflammatory parameters with age are observed for FLT3L and IL6, while for CSF1 and IL15, decreasing levels are associated with increasing age.

An increase in CXCL9 with age has been associated with various body processes, including immune dysregulation, metabolic changes in endothelial cells, immune signaling pathway dysregulation, and chronic inflammation, including low-grade inflammation (inflammaging) [34,78,79,80,81]. Due to this cytokine’s pro- and anti-inflammatory properties, its age-related increase may be associated with inflammaging, oxidative stress, hormonal and metabolic changes, and impaired regulation of signaling pathways influenced by health status and environmental factors [82,83,84,85].

The associations of inflammatory parameters with age and diseases identified by XAI (explainable AI) are described in more detail in Section 2.6.

The performance of the model for individual datasets is detailed presented in Supplementary Figure S2, for the majority of datasets characterized by a wide age range, the total MAE was in the range of 6–8 years.

EpInflammAge is quite competitive to existing immunological clock models, although it relies on predicted inflammatory parameters, rather than being directly measured. The iAge inflammatory clock has a higher average error of 15.2 years [34], the chronic renal disease-sensitive ipAGE clock model has a comparable mean absolute error of 7.27 years [38], the small immunological clock model SImAge also has a comparable mean absolute error of 6.94 years [35]. the CyClo age-related brain-atrophy-sensitive clock model showed a slightly lower mean absolute error of 6 years, but also has a lower correlation between the real and predicted age of about 0.5 [39]. EpInflammAge shows comparable results for inflammatory markers estimates, working originally on epigenetic data, thus bringing the epigenetic and inflammatory components of aging together.

So far, we evaluated the quality of the model’s performance on only healthy samples, without chronic or terminal diseases. However, it is challenging to assess its ability to detect age acceleration in patients with age-associated diseases and other pathological conditions.

### 2.4. Sensitivity of EpInflammAge and Other Epigenetic Clocks to Different Diseases

An important property of aging clock models (epigenetic, immunological and of another nature) is the sensitivity to diseases, such as the ability to detect age acceleration in patients with various pathologic conditions in comparison to healthy controls.

The relevant datasets from NCBI GEO are shown in Figure 1B. At this stage, we selected 52 out of 72, which contain both cases and controls, for the comparison to be possible. We do not consider cases-only datasets due to the heterogeneity of diseases and the inability to compare age acceleration with healthy controls under the same data collection conditions. The datasets give a fairly broad representation by disease group. A total of 13 ICD-11 chapters were found, and the number of individual disease codes within each chapter varied from 1 to 7. The outer ring of the chart shows which datasets contain samples with particular diseases (Figure 1B).

For infectious and parasitic diseases (Chapter 1), the model detects HIV and HIV-related groups quite well, showing an increase in age acceleration in patients compared to healthy controls (however, there are a few small datasets in which statistical significance is not observed, possibly due to small sample size or dataset specificity). Diseases of the immune system (Chapter 4) are poorly represented in the data; however, there was significant age acceleration in patients with systemic lupus erythematosus. Interestingly, mental, behavioral, and neurodevelopmental disorders (Chapter 6) were detected quite well by the model: for almost all datasets, statistically significant age acceleration was observed in patients with schizophrenia, psychosis, and depression. It is also noteworthy that the resulting age acceleration can yield different results for the same disease in different datasets (cf. HIV, Crohn’s disease). It could be the consequence of heterogeneity in the datasets in question, possibly due to their own batch effects and conditions of data collection and processing. The detailed results of the age acceleration analysis of the EpInflammAge model for controls and cases, grouped by ICD-11 chapters, are summarized in Supplementary Figure S3. The Mann–Whitney U-test compares two distributions (whether they are from the same population), and is often used to compare the statistical significance of the mean of two groups with a significance threshold; we used 0.05 (all *p*-values were FDR-adjusted according to the Benjamini–Hochberg procedure [86]). Because this test only applies to two distributions, we use it pairwise (apply it to each pair of groups, usually controls vs. cases). The *p*-value of the Mann-Whitney test indicates the presence or absence of statistical significance. The test does not, however, take into account the direction of change—that is, whether there is age acceleration or deceleration in one group relative to another. To assess the direction of change, the bias value should be considered. The latter is defined as the mean deviation of the predicted age from the actual age within a group; in this case, it is the mean age acceleration.

Next, we performed a large-scale comparison of EpInflammAge against 34 epigenetic clock models, shown with the development timeline in Figure 1C (for details cf. Methods, Section 4.3; model implementations were taken from the pyaging library). Most of the considered epigenetic models estimate age, and some of them estimate age-associated metrics (epiTOC1, ZhangMortality, DNAmTL, DunedinPACE, PCDNAmTL, stemTOC). As the benchmarks for age prediction, we made use of MAE and the correlation coefficient on controls (applicable only to those clocks that estimate age). Disease sensitivity is characterized by an index defined as the number of tests passed out of the total 76 (applicable to all models).

**Figure 5:**
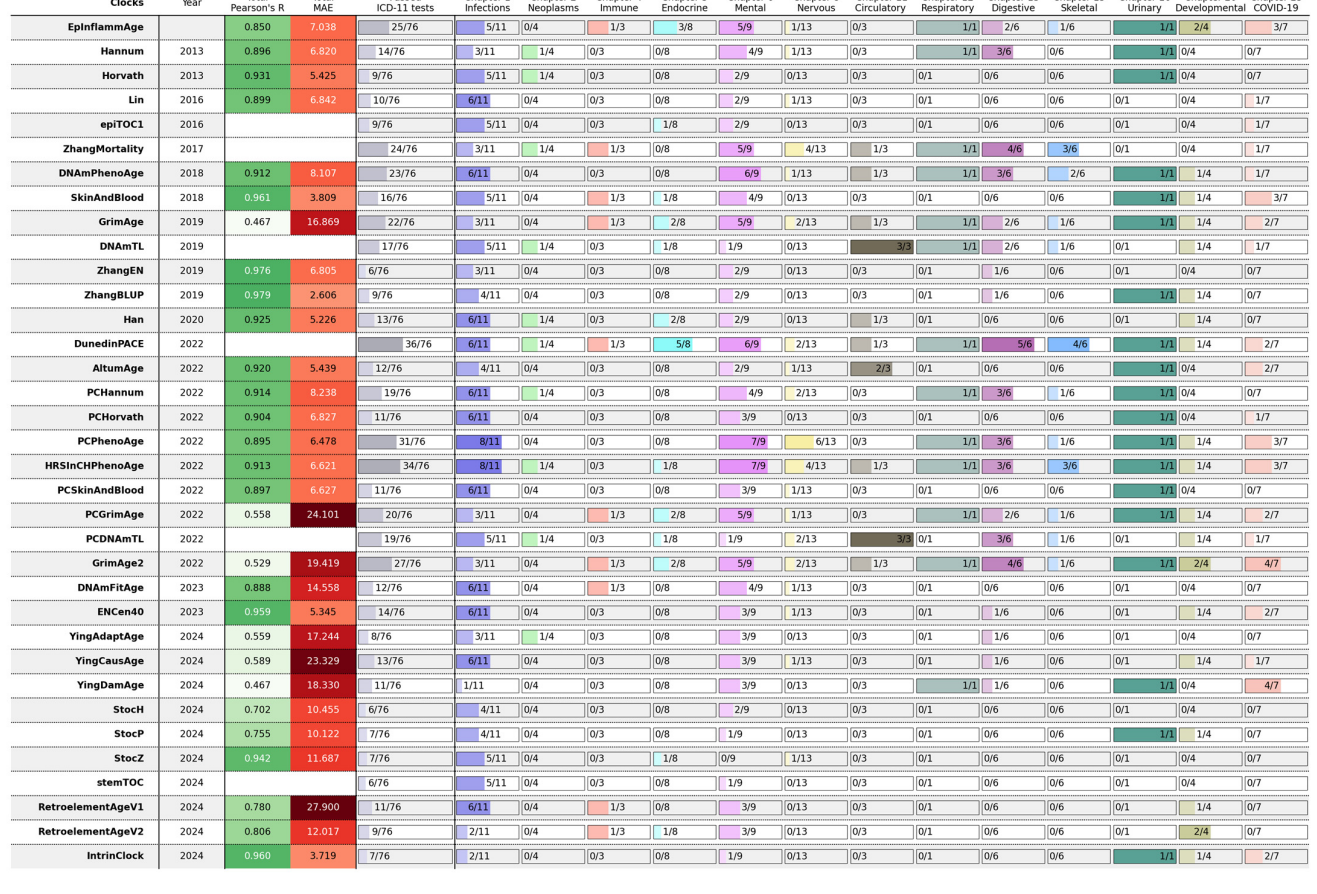
Comparative performance results of different epigenetic clocks (including the ones presented in this paper). The first column gives the names of the clocks. For each epigenetic clock model, the year of development (clocks are ordered by the chronology of their release), common metrics—Pearson correlation coefficient and MAE, and the number of disease sensitivity tests passed (Mann-Whitney *p*-value < 0.05) are given both overall and for each ICD-11 chapter. Pearson’s R and MAE values are additionally marked with color – darker colors indicate higher values for the corresponding metric.

Figure 5 summarizes the results. The best model in terms of quality metrics for healthy controls has proved to be ZhangBLUP (MAE has even less than 3 years, with a correlation coefficient over 0.97); however, it displays almost no disease sensitivity (only 9 tests out of 76 passed). The best model in terms of sensitivity to diseases is DunedinPACE (36 tests out of 76), which estimates the rate of aging rather than age. The HRSinCHPhenoAge and PCPhenoAge models (modifications of the DNAmPhenoAge model) perform quite well; they show high levels of age prediction metrics and have rather good sensitivity to diseases. Our EpInflammAge clock shows competitive results with the quality metrics of most popular models (19th by Pearson’s R, 15th by MAE on healthy controls), and rather high sensitivity to diseases (5th by number of tests passed). Unlike all other epigenetic models, it is also able to estimate inflammatory marker levels and interpret age acceleration in the inflammaging perspective, allowing them to extract more information from epigenetic data. There are disease groups where EpInflammAge outperforms most of the other clocks; e.g., for metabolic diseases (Chapter 5), only DunedinPACE passes more disease sensitivity tests but does not estimate age; for developmental anomalies (Chapter 20), models with similar disease group performance have worse control group quality metrics.

Overall, infectious, mental, respiratory, and genitourinary diseases (ICD chapters 1, 6, 12, 16, 25) are detected quite well by a large number of clock models, while neoplasms, immune, nervous, musculoskeletal, and developmental diseases (chapters 2, 4, 8, 15, 20) are detected poorly. There are several possible reasons for that. We already pointed out that methylation data can have a strong dependence on the laboratory conditions of the experiment, data preprocessing, and sampling characteristics. Besides, different chapters have different coverage by datasets, infectious, mental, and nervous diseases (chapters 1, 6, and 8) contain 11, 9, and 13 tests, respectively, while respiratory and genitourinary diseases (chapters 12 and 16) contain only 1. Further on, some diseases may not be reflected well by blood DNA methylation. For example, diseases of the nervous system are poorly detected (according to 11 tests). More detailed information on the sensitivity of different models to individual diseases is presented in Supplementary Figure S4. The results of the epigenetic models for the healthy controls are displayed in Supplementary Figure S5, and detailed results for all samples are listed in Supplementary Table S6. Thus, different models are good at detecting different groups of diseases, and such analysis is useful for selection of a suitable model.

Our EpInflammAge model shows comparable performance (in terms of MAE and Pearson’s R) to most epigenetic models on healthy controls. Models that perform significantly better on controls (with MAE of 2–3 years) are much less sensitive to disease. Also, one of the main advantages of our model is the ability to estimate inflammatory markers from epigenetic data in addition to assessing biological age and sensitivity to various diseases.

### 2.5. Applying XAI to the EpInflammAge Model

XAI has recently become an integral part of any AI-related research and development. It is a useful and powerful tool in the study of deep neural network models, allowing to open “black boxes”, which in fact are many modern AI models, since the principles of their decision-making are opaque for both developers and users. XAI approaches allow one to explain a model’s behavior from both the global (the general contributions of the model’s features to decision making) and the local (the influence of features on the model’s decision for each particular sample) perspectives, in order to track and correct model errors. There are many approaches that can be applied to different types of data, specialized for a particular class of models or applied to a wide range of architectures. In this paper, we used SHAP, a model-agnostic method based on the game theory idea that evaluates the contribution of each feature to the change in model prediction (cf. Methods, Section 4.2). For the developed EpInflammAge model, XAI was used to analyze the influence of inflammatory markers on relative age acceleration in the analysis of different pathologies, and to specify the particular contribution of each inflammatory marker, measured in years, to age acceleration.

**Figure 6:**
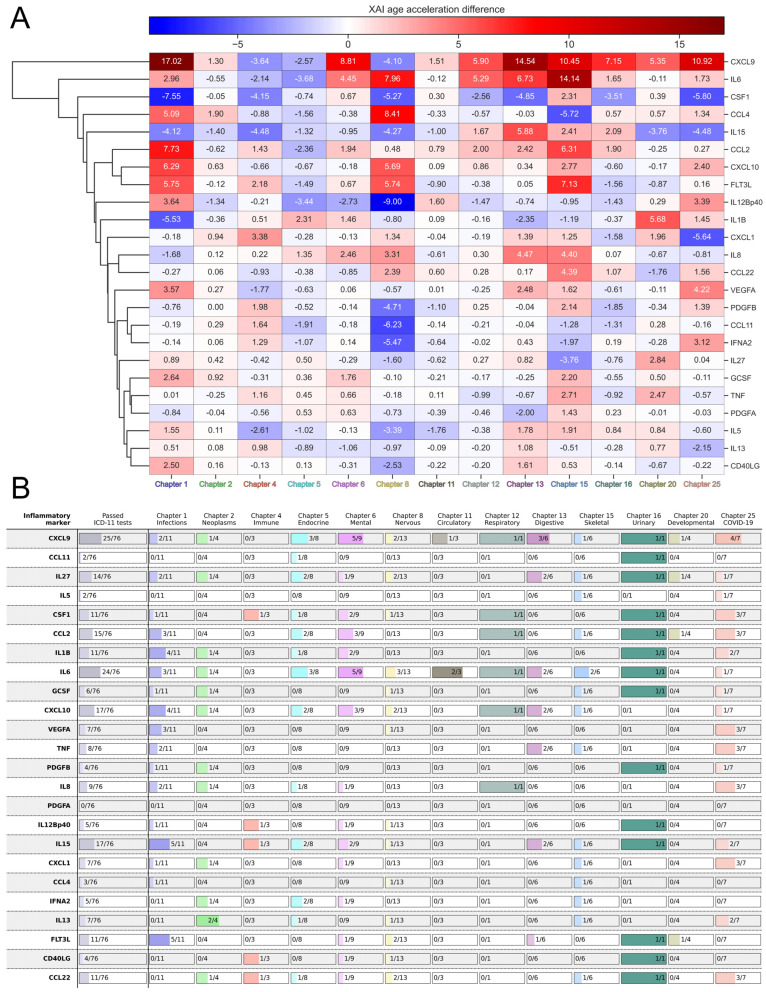
Association of selected inflammatory markers with different disease groups. (**A**) Cluster map illustrating the difference in age acceleration between cases and controls, determined using SHAP values, for all ICD-11 chapters considered. Positive values (red) correspond to higher values of age acceleration in cases compared to controls, while negative values (blue) correspond to lower values of age acceleration in cases compared to controls. (**B**) Comparative results of the association of different disease groups with the inflammatory markers studied. For each inflammatory marker, the number of disease sensitivity tests passed (Mann–Whitney *p*-value < 0.05) is given both overall and for each ICD-11 chapter.

Figure 6A shows a cluster map illustrating the difference in age acceleration determined by SHAP between cases and controls (positive values correspond to higher values of age acceleration in cases compared to controls, and negative values correspond to lower values). The biomarkers in the upper part of the cluster map—CXCL9, IL6, CSF1, CCL4, and IL15—have the greatest total effect on age acceleration. At the same time, some of them consistently contribute to age acceleration for most of the considered diseases (CXCL9, IL6), while some of them contribute to deceleration (CSF1, IL15). Note that there is not a single chapter for which all inflammatory markers have only positive or only negative contributions, nor is there a single inflammatory marker that shows only positive or only negative contributions for all disease chapters. For example, for diseases of the nervous system (Chapter 8), many markers display negative values, while for diseases of the musculoskeletal system (Chapter 15), many markers are positive.

Together with the results on the difference in age acceleration calculated via XAI, it is interesting to see how individual inflammatory markers are associated with different disease groups (Figure 6B). These results agree well with the cluster map, that CXCL9, IL6, and IL15 are the most sensitive to diseases. Chapters are detected differently by different inflammatory markers; the picture is generally worse than for the clocks. PDGFA does not detect any of the diseases. More detailed information on the sensitivity of different inflammatory markers to individual diseases is presented in Supplementary Figure S6.

Elevated CXCL9 levels in cardiovascular disease are associated with metabolic changes in endothelial cells, contributing to endothelial dysfunction [34,78]. CXCL9 serves as a biomarker of rheumatoid arthritis activity, regulated by IFN-γ and proinflammatory cytokines, which exacerbate synovial inflammation and joint damage [87]. Elevated levels of CXCL9 in mental disorders may arise from mechanisms involving interferon-γ-induced neuroinflammation, systemic inflammation, and inflammaging, which disrupt the integrity of the blood-brain barrier [80,88,89].

In rheumatoid arthritis, synovial fibroblasts and macrophages produce IL6, contributing to joint destruction, and chronic immune activation further contributes to IL6 overproduction [90,91]. In acute immune responses, including those to infectious diseases and cancer, NF-κB is activated in alveolar macrophages, causing overproduction of IL6, whose elevated levels trigger a cytokine storm [92,93]. Elevated IL6 in cardiovascular disease may be associated with activation of the inflammatory pathway, endothelial dysfunction, and epigenetic regulation [94,95,96,97].

### 2.6. EpInflammAge Web Interface

The EpInflammAge model for epigenetic-inflammatory age estimation is publicly available with a user-friendly interface [56]. The interface takes DNA methylation data for 2228 CpG sites (the list can be obtained from the web interface page) as input, and all samples must be assigned with age and have a unique identifier. Functionality allows us to process missing values in the data; one of three approaches can be chosen: kNN (k nearest neighbors), mean (filling missing values with mean values for a given CpG), and median (filling missing values with median values for a given CpG). The output of the interface provides the overall metrics, MAE and Pearson correlation coefficient, as well as a table with the results of the models: inflammatory marker estimates, EpInflammAge, and age acceleration values. In addition, interactive scatter and violin plots are provided, illustrating the relationship between chronological age and predicted age, and the distribution of age acceleration values, respectively.

Explainable artificial intelligence for the EpInflammAge model is also available in the provided web interface. For each individual sample (selected by unique identifier), an interactive waterfall plot can be obtained that shows the contribution of each individual feature to the final model prediction, and how they shift the predicted age relative to the chronological age. For each individual inflammatory marker, the levels relative to samples of similar age are given (what percentage of samples with similar chronological age have lower levels, and what percentage have higher levels). For the top three most important inflammatory markers, the correspondence age-associated diseases are outlined and supplied with references to the literature.

## 3. Discussion

We introduced EpInflammAge, a novel deep learning framework that bridges the epigenetic and inflammatory aspects of aging. Our results demonstrate three key advances: (1) successful prediction of inflammatory markers from DNA methylation data, (2) accurate age estimation using synthetic inflammatory profiles, and (3) robust disease sensitivity across multiple pathological conditions. These findings have important implications for both aging research and clinical applications. The model is accessible via a web interface, enabling users to retrieve results (inflammatory parameter levels and age estimates) with minimal delay and perform XAI interpretation for each sample.

One of the primary objectives of this research was to integrate the two hallmarks of aging—namely, epigenetic modifications and immunosenescence. To this end, we conducted a simultaneous examination of DNA methylation data and levels of cytokines and chemokines. We developed models for estimating inflammatory marker levels from epigenetic profiles and subsequently evaluated their performance on a large cohort of healthy and diseased samples. As measuring inflammation is clinically significant, the developed model enables the acquisition of epigenetic data and the prediction of inflammatory biomarkers based on methylation. This development presents an opportunity to progress in the direction of evaluating inflammaging, which is characterized by low-grade inflammation associated with age and age-related diseases.

Estimates of inflammatory markers have proven to be most sensitive to infectious and parasitic diseases, including COVID-19, as well as respiratory and genitourinary diseases. The results of studies [98,99,100,101] indicate that IL15, FLT3L, CXCL10, and IL1B, which demonstrated the most promising outcomes among the estimates, are actually associated with numerous viral, bacterial, parasitic, and fungal infections. Additionally, one of the most extensively studied diseases in recent years, COVID-19 and its associated complications, have been shown to induce alterations in the levels of the majority of the studied inflammatory markers [102,103,104,105,106,107,108,109]. CXCL9, CCL2, IL6, CXCL10, and IL8 have been linked to various respiratory diseases [110,111,112,113,114] and retain sensitivity for their level estimates as well. Chronic kidney disease, which has a significant impact on the body’s immune system and is associated with irreversible changes in cytokine levels [38,115,116,117,118,119,120], also causes notable alterations in the levels of the majority of synthetic inflammatory markers.

The estimates of inflammatory markers have proven to be least sensitive to diseases of the immune, cardiovascular, and musculoskeletal systems, as well as developmental anomalies. This may be attributed to the limited representation of these diseases in the analyzed data. The estimation of inflammatory parameters is based on a limited number of CpG sites, which may result in the omission of certain aspects of cytokine functionality, which could also be a contributing factor to the reduced sensitivity. Low sensitivity observed with respect to diseases of the nervous system may be attributed, at least in part, to the anatomical location of these diseases, which are frequently confined to the central nervous system and separated from the blood with the blood-brain barrier. Consequently, these diseases may be manifested less conspicuously in blood parameters.

The estimated levels of CXCL9 and IL6 demonstrate sensitivity to almost all disease groups, which is consistent with their important role in the inflammatory response in humans and their association with multiple age-related and pathological changes [102,110,115,121,122,123,124,125,126,127,128,129,130]. In conclusion, there is compelling evidence that estimates of inflammatory markers mirror associations between their real-world counterparts and various disease groups. However, further investigation is necessary, given the limited scope of diseases represented in the current study.

The model estimating biological age has several requirements: the model should work well for healthy controls (demonstrate low error in predicted age and high correlation with chronological age), the model should also be sensitive to different diseases (demonstrate significant age acceleration or deceleration in cases compared to healthy controls), and it may have additional advantages. The EpInflammAge model performs on healthy controls comparably to most analyzed epigenetic models, it is sensitive to different diseases (better than epigenetic models that perform exceptionally well for healthy controls), and it estimates inflammatory markers in addition to biological age. It is important to note that ElasticNet—despite its prevalence as a methodology for constructing epigenetic clock models, or its linear nature—proved to be less effective in this particular task when compared to modern deep neural network architectures designed for tabular data.

It should also be noted that there is a paper with a comparable training sample size that utilizes a similar methodology for estimating biological age (InflammAge) [52]. It proposes the estimation of inflammatory markers (EpiScores) from methylation data, and then estimates biological age from those levels. However, it is important to note several significant differences. Specifically, EpiScores are predicted on a non-interpretable scale (in this study, we predict inflammatory biomarker levels in standard units of measurement—pg/mL), resulting in lower average correlation coefficients between measured and estimated inflammatory marker values. The study [52] utilizes a linear ElasticNet, which we demonstrate can be surpassed by deep neural networks. Furthermore, it should be noted that calculating InflammAge requires a request to the paper’s authors. We have developed a user-friendly publicly accessible web interface for our EpInflammAge model.

The use of a large amount of epigenetic data for controls and cases allowed us not only to build a model of age estimation and test its sensitivity to diseases, but also to conduct a direct large-scale comparison with other epigenetic clock models. We inferred the consistency of the models, investigated their applicability to different groups of diseases, compared their performance on cases and controls, confirmed the associations found earlier, and identified new associations.

Clock models that are frequently used in the field have previously been tested on a wide range of data in multiple studies, and this analysis confirmed associations with a wide range of diseases, to name the Hannum [25,31,32,33,131,132], Horvath [24,31,32,33,38,131,133,134], DNAmPhenoAge [26,31,38,131,133,134,135,136], and GrimAge clocks [27,31,33,38,131,135,136,137,138,139,140], as well as DunedinPACE [29,30,132,141,142,143,144,145,146,147].

The sensitivity of many clock models to diseases of the immune, digestive, and genitourinary systems, as well as developmental anomalies, has been understudied, and this study aims to bridge this gap. The EpInflammAge model, which combines inflammatory and epigenetic components with several epigenetic clock models (ZhangMortality and DunedinPACE for arteritis; SkinBlood, both GrimAge, both RetroElementAge for lupus), is able to detect immune system diseases. In the majority of cases, Crohn’s disease and ulcerative colitis (diseases of the digestive system) are either recognized by the models simultaneously or not at all. Instructively, it was found that chronic kidney disease, which is associated with changes in many inflammatory marker estimates, results in significant alterations according to the majority of epigenetic clock models. Furthermore, it appears that profound changes in stage 5 disease are also reflected in the epigenetic profile. The low sensitivity of the clock to developmental abnormalities can be attributed to the rarity of these pathologies, their varied manifestations, and the fact that they may not always align with the genomic regions utilized in the model’s construction.

It is worth noting that the EpInflammAge model has proven to be the only one to show sensitivity to diabetes, and stands among the best in its sensitivity to a number of other conditions, including systemic lupus erythematosus, psychosis, depression, hypopituitarism, Creutzfeldt-Jakob disease, acute respiratory distress syndrome, chronic renal failure, CHARGE and Kabuki syndromes, as well as COVID-19. The model’s differential sensitivity across diseases may be influenced by limited statistical power for specific conditions, the inherently weaker signal of localized pathologies (e.g., disorders of nervous system) in blood-derived markers, the model’s focus on inflammatory pathways, and the clinical heterogeneity of complex diseases.

The proposed analysis can be particularly useful in the case of epigenetic clock models that have only recently been developed. There is still a lack of studies on different cohorts, and testing sensitivity to a wide range of diseases is a priority. It is noteworthy that the AdaptAge, CausAge, and DamAge models, which are based on a similar principle, exhibit significant differences in their sensitivity to infectious diseases. Stochastic versions of the Horvath, PhenoAge, and Zhang clocks demonstrate varying degrees of sensitivity to mental disorders. Additionally, the RetroElementAge model shows promise in detecting atypical aging patterns associated with developmental abnormalities.

### Limitations

Despite EpInflammAge’s strong performance, several key limitations must be considered when interpreting results and planning future applications:

Sample Size Constraints: The inflammatory embedding is based on a relatively modest sample size (n = 329), which may limit the model’s ability to capture the full spectrum of inflammatory profile variations. This may also affect the correlation between predicted and actual values of inflammatory markers, the absolute values of which are less than 0.6. The model has been trained on data sets that include not only healthy controls, but also patients with ESRD, which may influence the model’s results. Nevertheless, the models have been trained on a broader spectrum of inflammatory values, which may enhance their capacity for generalization. The results can also be affected by an imbalance in the number of samples between the two steps of model construction. This limitation is closely related to the difficulty in obtaining the necessary data used to construct the model. Specifically, DNA methylation data, inflammatory profiles, and phenotype information (such as sex, age, and diseases) for the same participants must be available simultaneously. There is currently no publicly available data of this type.

Data Heterogeneity: Epigenetic data from different datasets exhibit substantial technical variation and batch effects, potentially impacting model generalizability. The results may be influenced by a variety of factors, including but not limited to: (1) phenotypic characteristics, such as nationality/geographical origin of the samples, sex, and age distribution; (2) lifestyle characteristics, such as diet, physical activity, harmful habits, and stress; (3) socio-cultural characteristics, such as wealth, access to medical care, and education level; (4) many other factors. There are also several technologies available for acquiring immunological/inflammatory data. Further study is necessary to determine the applicability of the developed model to data collected by methods other than Luminex.

Data Source: The EpInflammAge model is blood-based. Including a wider range of tissues could enhance the model’s robustness and expand its applicability to different groups of pathologies.

Disease Representation: The availability of certain disease groups in public repositories is limited, affecting our ability to comprehensively assess model performance across all pathological conditions.

Feature Selection Methodology: While our mRMR-based approach proved effective, alternative feature selection strategies might yield different or potentially improved marker sets.

Model Architecture Choices: Though we employed state-of-the-art deep learning architectures, the rapidly evolving nature of AI suggests that newer approaches may offer further improvements.

Future work should address these limitations through larger-scale validation studies and exploration of emerging methodologies.

## 4. Materials and Methods

### 4.1. Data Collection and Processing

#### 4.1.1. Concurrent Data from Inflammatory and Epigenetic Profiles

Initial model development utilized a proprietary dataset comprising paired inflammatory and epigenetic profiles collected between 2019–2023, encompassing both healthy controls and disease states. It included: healthy samples from Russia (223 participants aged 19 to 101 years), samples with ESRD from the Nizhny Novgorod region enrolled by “FESPHARM NN” hemodialysis centers (106 participants aged 25 to 88 years). The peculiarities of the study procedure, all possible inconveniences, and risks were explained to all participants. Each participant filled in the consent for personal data processing and signed their informed consent, taking into account the principle of confidentiality (availability of personal data only to the research team and presentation of data in a common array). The study was approved by the local ethical committee of Nizhny Novgorod State University. All procedures were in accordance with the Declaration of Helsinki 1964 and its later amendments.

To obtain inflammatory profiles, the analysis was performed on plasma using the K3-EDTA anticoagulant, without hemolysis and lipemia. Plasma was thawed, spun (3000 rpm, 10 min) to remove debris, and 25 µL was collected in duplicate. Plasma with antibody-immobilized beads was incubated with agitation on a plate shaker overnight (16–18 h) at 2–8 °C. The Luminex^®^ assay was run according to the manufacturer’s instructions, using a custom human cytokine 46-plex panel (EMD Millipore Corporation, Darmstadt, Germany, HCYTA-60 K-PX48). Assay plates were measured using a Magpix (Milliplex MAP). Data acquisition and analysis were performed using the standard MAGPIX® software program set xPONENT version 4.2. Data quality was examined based on the following criterion: the standard curve for each analyte has a 5P R2 value > 0.95. To pass assay technical quality control, the results for two controls in the kit needed to be within 95% of the CI (confidence interval) provided by the vendor for >40 of the tested analytes. No further tests were performed on samples with results out of the low range (<OOR). Samples with results out of the high range (>OOR) or greater than the standard curve maximum value (SC max) were not tested at higher dilutions.

To obtain epigenetic profiles, Phenol Chloroform DNA extraction was used. DNA was quantified using the DNA Quantitation Kit Qubit dsDNA BR Assay (Thermo Fisher Scientific, Waltham, MA, USA), and 250 ng was bisulfite-treated using the EpiMark Bisulfite Conversion Kit (New England Biolabs, Ipswich, MA, USA) with case and control samples randomly distributed across arrays. The Illumina Infinium MethylationEPIC BeadChip (San Diego, CA, USA) [148] was used according to the manufacturer’s instructions. DNA methylation was expressed as beta values, ranging from 0 for unmethylated to 1 representing complete methylation for each probe. DNAm data preprocessing and normalization were performed with the standard pipeline in the ChAMP R package (version 2.38.0) [149]. During preprocessing, probes with a detection *p*-value above 0.01 in at least 10% of samples were removed. Functional normalization of raw methylation data was performed using the minfi R package (version 1.54.1) function [150].

#### 4.1.2. GEO

We used the R packages GEOmetadb (version 1.70.0) [151] and GEOquery (version 2.76.0) [152] to perform a preliminary analysis of all available datasets in the GEO repository [59]. These tools give access to metadata related to samples, platforms, and datasets. The Python package GEOparse (version 2.0.4) [153] was used to retrieve features and fields in the datasets, and in some cases to automatically download pre-processed methylation data.

To train the EpInflammAge epigenetic-inflammatory clock model, we selected data from healthy controls only. To test the models’ sensitivity to diseases, we selected datasets that contain both cases and controls to examine whether there is age acceleration in patients with diseases compared to healthy controls. All samples are required to have age and sex information. This is necessary for model training as well as for calculating estimates of other epigenetic models and age acceleration. Functional normalization of raw methylation data (if available) was performed using the minfi R package function [150].

### 4.2. Model Development and Architecture

#### 4.2.1. Feature Selection

The epigenetic data have a huge dimensionality (Illumina 450k and Illumina EPIC standards overlap in 400,000 CpG sites), and only a part of them are associated with certain parameters, so we performed feature selection before model training. Feature selection also helps to reduce model training time, improve quality metrics by reducing the number of noisy features, and reduce the risk of overfitting. At the first step of estimating inflammatory feature levels from epigenetic data, we used the mRMR (minimum Redundancy–Maximum Relevance) approach for feature selection, which was first proposed in the context of gene expression data [154]. mRMR is a filter method that differs from the wrapper or embedded methods (like Lasso or ElasticNet) in its approach to reducing redundancy. It is known that “N best features are not the best N features” [155], because important features can be correlated and redundant. The mRMR approach ranks features based on their importance in predicting the target variable (in our case, inflammatory marker level), with importance necessarily taking into account redundancy and relevance. The relevance of each individual feature is estimated based on the f-statistics with the target variable, redundancy takes into account the correlation of a new feature with the selected ones in the previous iterations (if a new feature is highly correlated with the already selected ones, it is unlikely to provide much new information). The mRMR approach works iteratively, selecting one new feature at a time. At the first step, 100 CpG sites for each inflammatory marker were selected using mRMR to build models for estimating inflammatory marker levels from epigenetic data. A total of 2936 CpG sites were used to estimate all 32 inflammatory parameters (all CpGs are listed in Supplementary Table S2). During model development, different numbers of CpG sites were used for each inflammatory marker (100, 200, 300 and more); however, increasing the number of features did not improve the model’s metrics (mainly the correlation coefficient). Therefore, we chose to use 100 CpG sites to maintain a balance between the number of features and model performance (not too many CpG sites for good performance).

At the second step of age prediction from inflammatory estimates, feature selection is also used. Due to their small number, the Pearson correlation coefficient with a threshold of 0.5 is used here—only inflammatory marker estimates correlating with their real values are used for age prediction (for example, the GrimAge model uses a threshold of 0.35). Thus, 24 inflammatory markers are used to estimate age, which, in turn, are estimated from 2228 CpG sites.

#### 4.2.2. Models Training

At the first step, a separate model is built for each inflammatory parameter. It is important to note that the model does not predict the actual levels of inflammatory indicators, but rather the log-transformed values. Transforming the target variable (in this case, inflammatory marker levels) is often used in data with large variance to reduce skewness, bringing the distribution of the data closer to normal, and improves the stability of gradient flow in backpropagation. This transformation can significantly improve the performance of machine learning models. Original inflammatory marker values can be obtained using inverse transformation.

Model development employed a classical linear ElasticNet approach and contemporary deep neural network architectures optimized for tabular data analysis, including Multi-Layer Perceptron (MLP), Deep Abstract Network (DANet) [156], Feature Tokenizer and Transformer (FT-Transformer) [157], and Gated Adaptive Network for Deep Automated Learning of Features (GANDALF) [158]. Unlike sequential or image data, tabular data lacks inherent spatial or temporal relationships between features, necessitating specialized architectural considerations. Epigenetic and inflammatory data are tabular, as they are a set of numerical values for each sample. MLP is one of the simplest neural network architectures, consisting of several dense layers. FT-Transformer is an adaptation of the Transformer architecture to tabular data, GANDALF uses a new tabular processing unit called the Gated Feature Learning Unit (GFLU) with a gating mechanism and built-in feature selection, and DANet is focused on abstract layers whose main idea is to group correlated features and create higher-level abstract features from them.

Before training the models, all data was divided into two parts—80% for training/validation and 20% for independent testing. During model training, cross validation was used—sequential division of data into training and validation subsets at a ratio of 3 to 1, until all data have been in the role of training and validation. Cross-validation is necessary to determine the best combination of model hyperparameters that provide the best metrics.

The software implementation of hyperparameter optimization was taken from the optuna python package (version 2.10.1) [159], using the Tree-structured Parzen Estimator (TPE) algorithm [160]. TPE is an iterative process that uses the history of evaluated hyperparameters to create a probabilistic model that is used to propose the next set of hyperparameters for evaluation. The total number of optimization trials for each considered model was set to 1024. The number of random sampled startup trials was set to 256. The number of candidate samples used to calculate the expected improvement was set to 16. Supplementary Table S7 lists the varied hyperparameters for each model with a range of variation.

The final model results were computed on a test subset (not involved in model training at all). To evaluate the quality of the models, we consider the following metrics: MAE (mean absolute error), and Pearson correlation coefficient.

The second step also uses ElasticNet and the same neural network architectures with cross-validation and hyperparametric search. Stratification of samples by age was also used here—in each dataset, all samples were divided into 5 quintiles (groups) by age, and each group must contain at least 5 samples. If this condition cannot be met (the dataset is too small to provide at least 5 samples in each group), it is placed completely in the test data group. The results for all architectures at both steps are summarized in Supplementary Table S3 and Supplementary Figure S1. Age acceleration was calculated as the difference between predicted and real age for each sample.

#### 4.2.3. XAI

Deep neural network architectures are often black boxes with non-transparent decision-making principles. This complicates both the process of tracking model errors and understanding the reasons why models make certain decisions. However, at present, explainable artificial intelligence approaches are being actively developed that can explain why models predict certain values, in particular when solving a regression problem. One of the most common approaches are SHAP values [161], which use game theory principles. They show how a particular value of each feature changes the final model prediction for each sample. As a result, they help identify the features that contribute most to the final model age prediction.

### 4.3. Statistical Analysis and Validation

#### 4.3.1. Statistical Tests

One of the stages of clock model analysis is to check their association with different diseases. The distributions of age acceleration values between the different considered groups (controls and different types of cases) were tested using the Mann–Whitney U-test [162]. This is a nonparametric test for comparing the results of two independent groups, and is used to test the probability that two samples come from the same population, with a two-sided null hypothesis that the two groups are not the same. Statistical significance was assessed using multiple testing corrections with the Benjamini-Hochberg procedure to control the false discovery rate (FDR) with a 0.05 threshold. All reported *p*-values are FDR-adjusted unless otherwise specified [86]. Additionally, a bias value was calculated to estimate the direction of change (acceleration or deceleration) as the mean deviation of predicted values from real values. In the case of age, this is referred to as age acceleration.

#### 4.3.2. Epigenetic Clock Models

The implementations of existing epigenetic clock models against which the EpInflammAge model was compared were taken from the pyaging library, which aggregates a large number of clock models for different types of data, organisms, and tissues [163]. For inclusion in the current work, we selected human epigenetic clock models that estimate both age and age-associated metrics and can be applied to a wide age range (in particular, clocks that work only for newborns or children, as well as for centenarians, were excluded). A total of 34 models of epigenetic clocks were selected for comparative analysis: Hannum [25], Horvath [24], Lin [164], epiTOC1 (mitotic-like clock, estimates time of cancer) [165], ZhangMortality (estimates mortality risk) [166], DNAmPhenoAge [26], SkinAndBlood [167], GrimAge [27], DNAmTL (estimates telomere length) [168], ZhangEN, ZhangBLUP [169], Han [170], DunedinPACE (estimates pace of aging) [29], AltumAge [171], PCHannum, PCHorvath, PCPhenoAge, HRSInCHPhenoAge, PCSkinAndBlood, PCGrimAge, PCDNAmTL (PC modifications of corresponding epigenetic clock models) [172], GrimAge2 [173], DNAmFitAge [174], ENCen40 [175], YingAdaptAge, YingCausAge, YingDamAge [30], StocH, StocP, StocZ (stochastic versions of Horvath, DNAmPhenoAge, ZhangMortality epigenetic models) [176], stemTOC (stochastic mitotic-like clock, estimates time of cancer) [177], RetroelementAgeV1, RetroelementAgeV2 [178], and IntrinClock [179]. Imputation of missing values was performed using the kNN method.

#### 4.3.3. GSEA

We merged the CpG sites that were selected for models of inflammatory parameters estimation and identified their corresponding genes, 1560 genes in total (Supplementary Table S4). For these genes, we performed gene-set enrichment analysis using the Enrichr library [60,61,62]. This library contains more than 500 thousand terms from 230 libraries. All libraries in Enrichr are organized into 8 groups according to the topics of gene sets and terms: Transcription, Pathways, Ontologies, Diseases/Drugs, Cell Types, Misc, Legacy, and Crowd. Groups Misc (miscellaneous) and Legacy (earlier, already out-of-date versions of libraries) were not considered. We also removed from consideration all libraries and terms related to other organisms (not humans). The resulting list of terms was filtered by adjusted *p*-value with a threshold of 0.001 and resulted in 314 terms being included (Supplementary Table S5).

### 4.4. Web Interface

The web interface for the EpInflammAge model is based on the Gradio framework [180], which helps to create user-friendly applications to facilitate the use of machine learning models. The application supports imputation of missing values in the uploaded data (kNN, mean, median), inference of trained models estimating age and inflammatory levels, and application of XAI (SHAP) for local explainability of the model result for each sample. To make the model available to a wide community of users, it is hosted on HuggingFace, one of the largest platforms for hosting AI models, datasets, and interfaces for community spaces.

## Data Availability

The data used to construct the models are given in the Supplementary Tables. Open-access DNA methylation data were downloaded from the NCBI GEO repository, all corresponding GSE codes are summarized in Figure 1 and GSM codes in Supplementary Table S6. The web interface for using the EpInflammAge model is available at the following link: https://huggingface.co/spaces/UNNAILab/EpInflammAge (accessed on 27 June 2025). The source code for the pipeline presented in this paper is publicly available in the repository: https://github.com/GillianGrayson/EpInflammAge (accessed on 27 June 2025).

## Supplementary Materials

The supporting information can be downloaded at: https://www.mdpi.com/article/10.3390/ijms26136284/s1.

## Abbreviations

The following abbreviations are used in this manuscript:

AI	Artificial Intelligence
COPD	Chronic Obstructive Pulmonary Disease
COVID-19	Coronavirus Disease 2019
DANet	Deep Abstract Network
DNA	Deoxyribonucleic Acid
FT-Transformer	Feature Tokenizer and Transformer
GANDALF	Gated Adaptive Network for Deep Automated Learning of Features
GEO	Gene Expression Omnibus
GFLU	Gated Feature Learning Unit
GO	Gene Ontology
GSEA	Gene-Set Enrichment Analysis
ICD	International Classification of Diseases
HIV	Human Immunodeficiency Virus
KDE	Kernel Density Estimation
KEGG	Kyoto Encyclopedia of Genes and Genomes
kNN	k Nearest Neighbors
MAE	Mean Absolute Error
mRMR	Minimum Redundancy, Maximum Relevance
RNA	Ribonucleic Acid
SHAP	Shapley Additive Explanations
XAI	Explainable Artificial Intelligence
