# Supplementary material for: EpInflammAge: Epigenetic-Inflammatory Clock for Disease-Associated Biological Aging Based on Deep Learning
Source: Int J Mol Sci. 2025 Jun 29;26(13):6284. doi: 10.3390/ijms26136284 (PMC12249966; doi:10.3390/ijms26136284)
Supplement: Supplementary file 1 [file ijms-26-06284-s001.zip › Figure captions.docx]

**SupplementaryFigureS1.pdf**. Comparison of Pearson correlation coefficients between actual and predicted values for all tested neural network architectures and all constructed models.

**SupplementaryFigureS2.pdf**. EpInflammAge results for all considered datasets. For each dataset, the relationship between real and predicted age is presented, KDE corresponds to train-validation data, scatter corresponds to test data (if the dataset is too small, it only participates in the test data). The table for each dataset shows MAE values and Pearson correlation coefficient total and separately for train, validation, test data.

**SupplementaryFigureS3.pdf**. Age acceleration estimated by the EpInflammAge model for controls and cases for different disease groups. Statistical significance of the difference in mean values of age acceleration was assessed pairwise using the Mann-Whitney U-test with an FDR-adjusted p-value threshold of 0.05 (76 tests in total). Different backgrounds correspond to different chapters of ICD-11. Each plot shows the violins of age acceleration in different groups, with the name of the corresponding dataset (GEO code) and the number of samples in it. The name of the group (control - healthy participants, or cases - patients with different diseases), the number of samples in the group and bias value (mean age acceleration within a group) are given under each violin. FDR-adjusted p-values are given between the violins.

**SupplementaryFigureS4.pdf**. Detailed performance results of different epigenetic clocks (including the one presented in this paper). The first column gives the names of the clocks. For each epigenetic clock model, the year of development (clocks are ordered by the chronology of their release), common metrics - Pearson correlation coefficient and MAE, and the number of disease sensitivity tests passed (Mann-Whitney p-value < 0.05) are given both overall and for each ICD-11 disease code.

**SupplementaryFigureS5.pdf**. Dependence of the predicted age on the real age for different epigenetic clocks (gray KDE) with the corresponding linear regression (red line).

**SupplementaryFigureS6.pdf**. Detailed results of the association with different diseases of the inflammatory markers studied. For each marker, the number of disease sensitivity tests passed (Mann-Whitney p-value < 0.05) is given both overall and for each ICD-11 code.
