# Supplementary material for: EpInflammAge: Epigenetic-Inflammatory Clock for Disease-Associated Biological Aging Based on Deep Learning
Source: Int J Mol Sci. 2025 Jun 29;26(13):6284. doi: 10.3390/ijms26136284 (PMC12249966; doi:10.3390/ijms26136284)

Chapter 1: Certain infectious or parasitic diseases

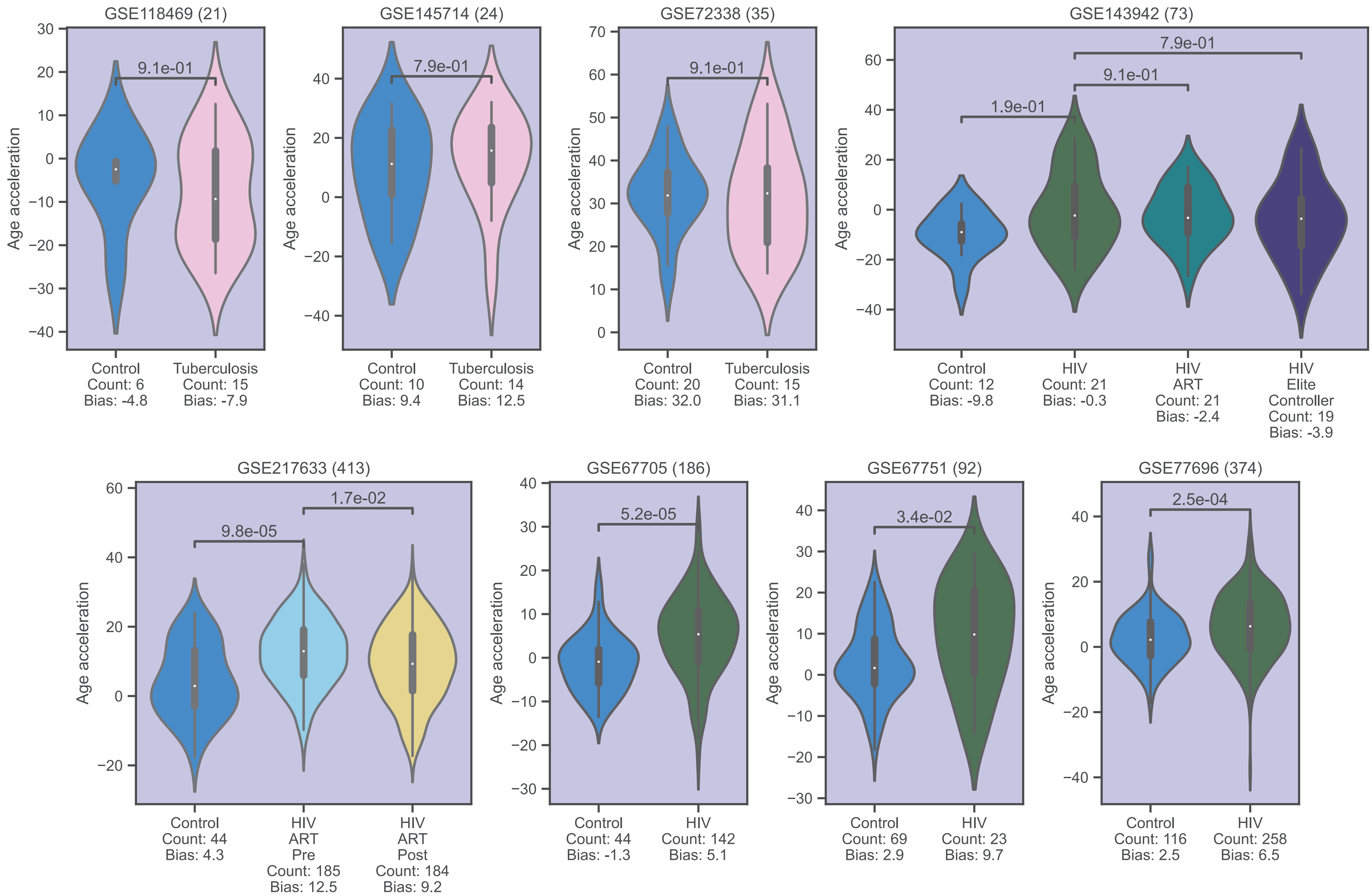

Chapter 2: Neoplasms

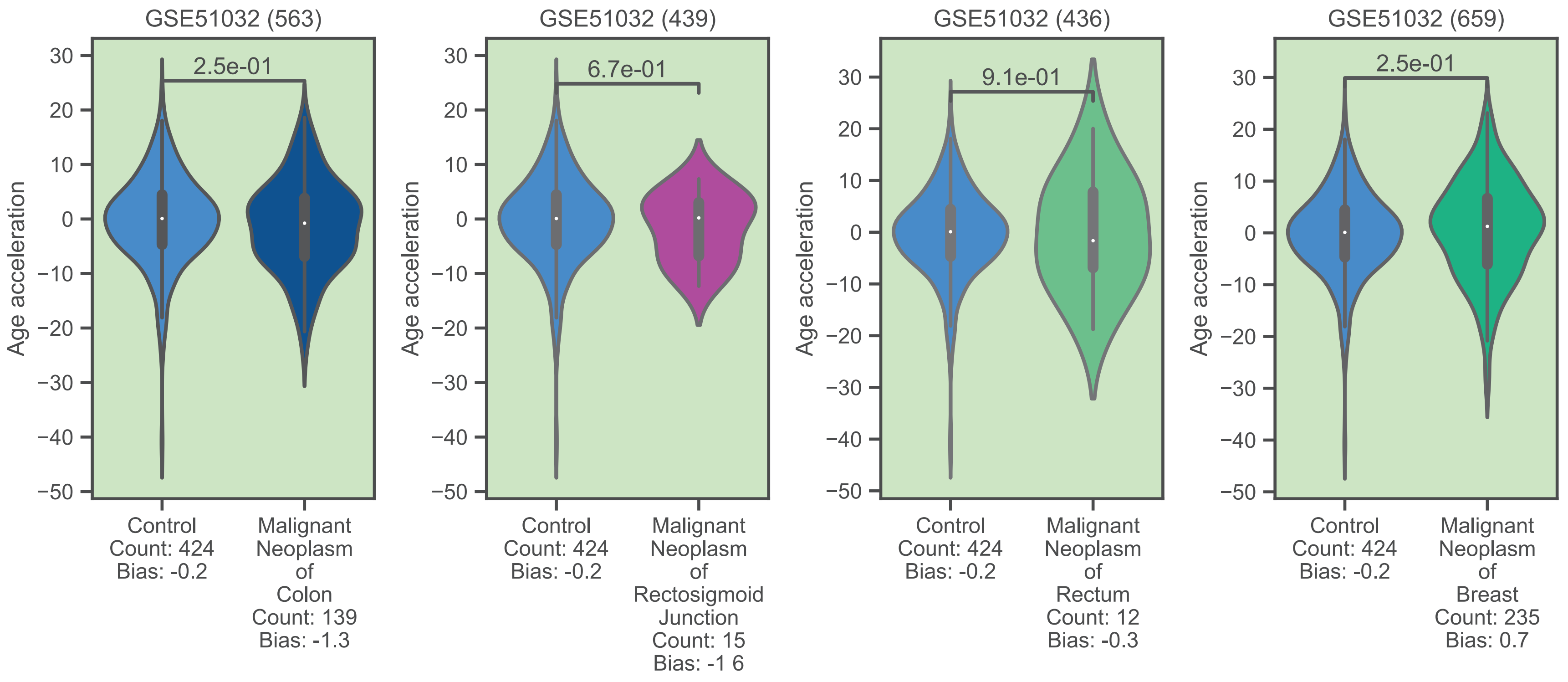

Chapter 4: Diseases of the immune system

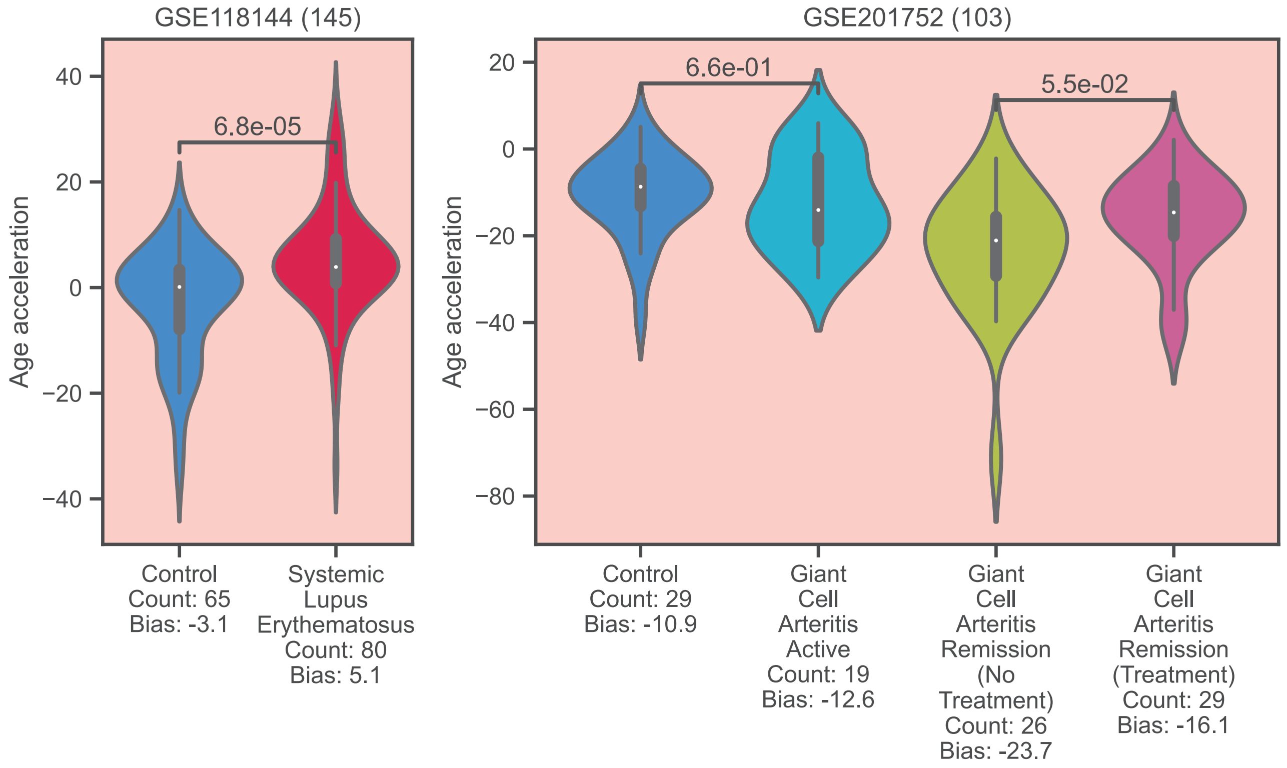

Chapter 5: Endocrine, nutritional or metabolic diseases

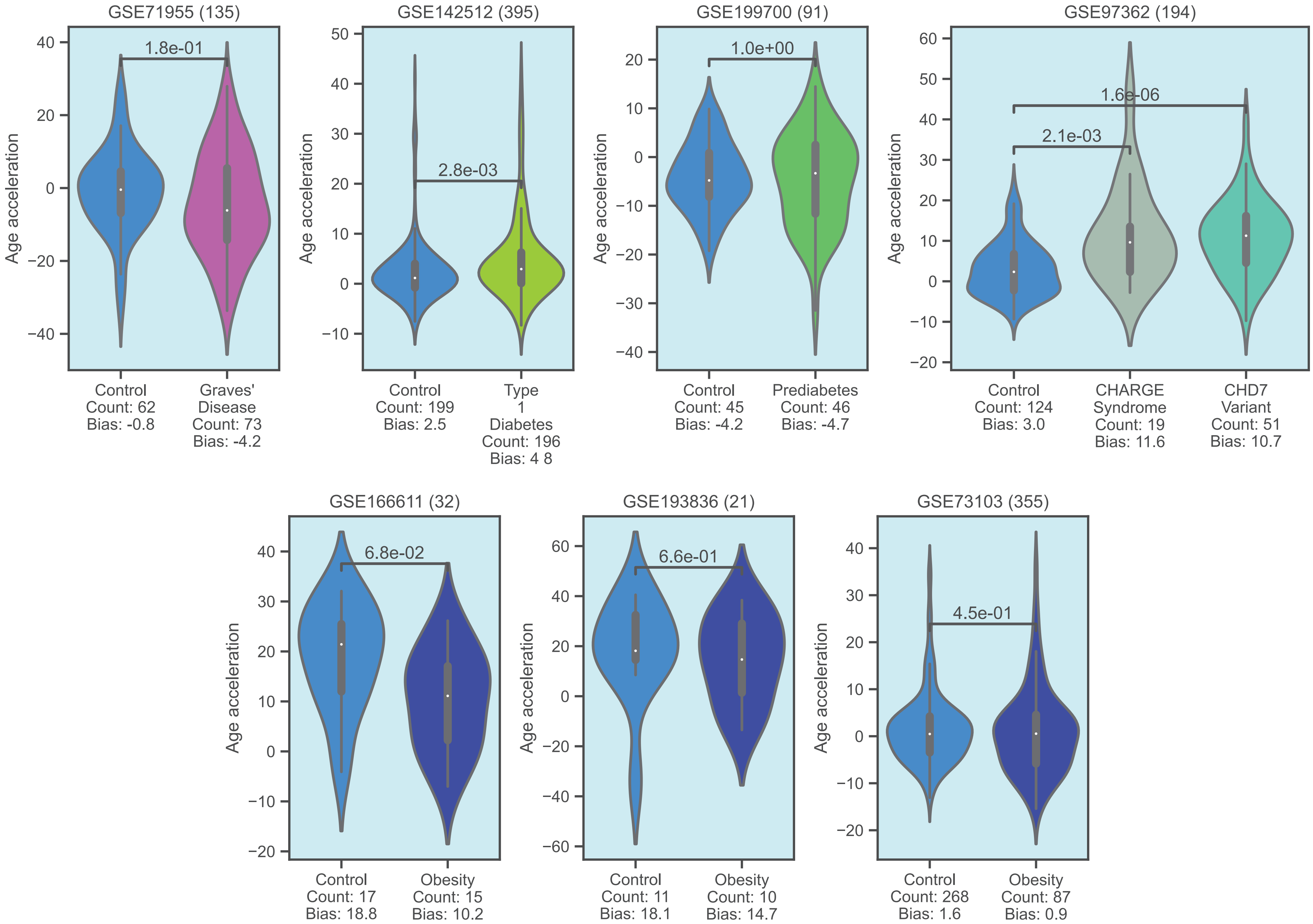

Chapter 6: Mental, behavioural or neurodevelopmental disorders

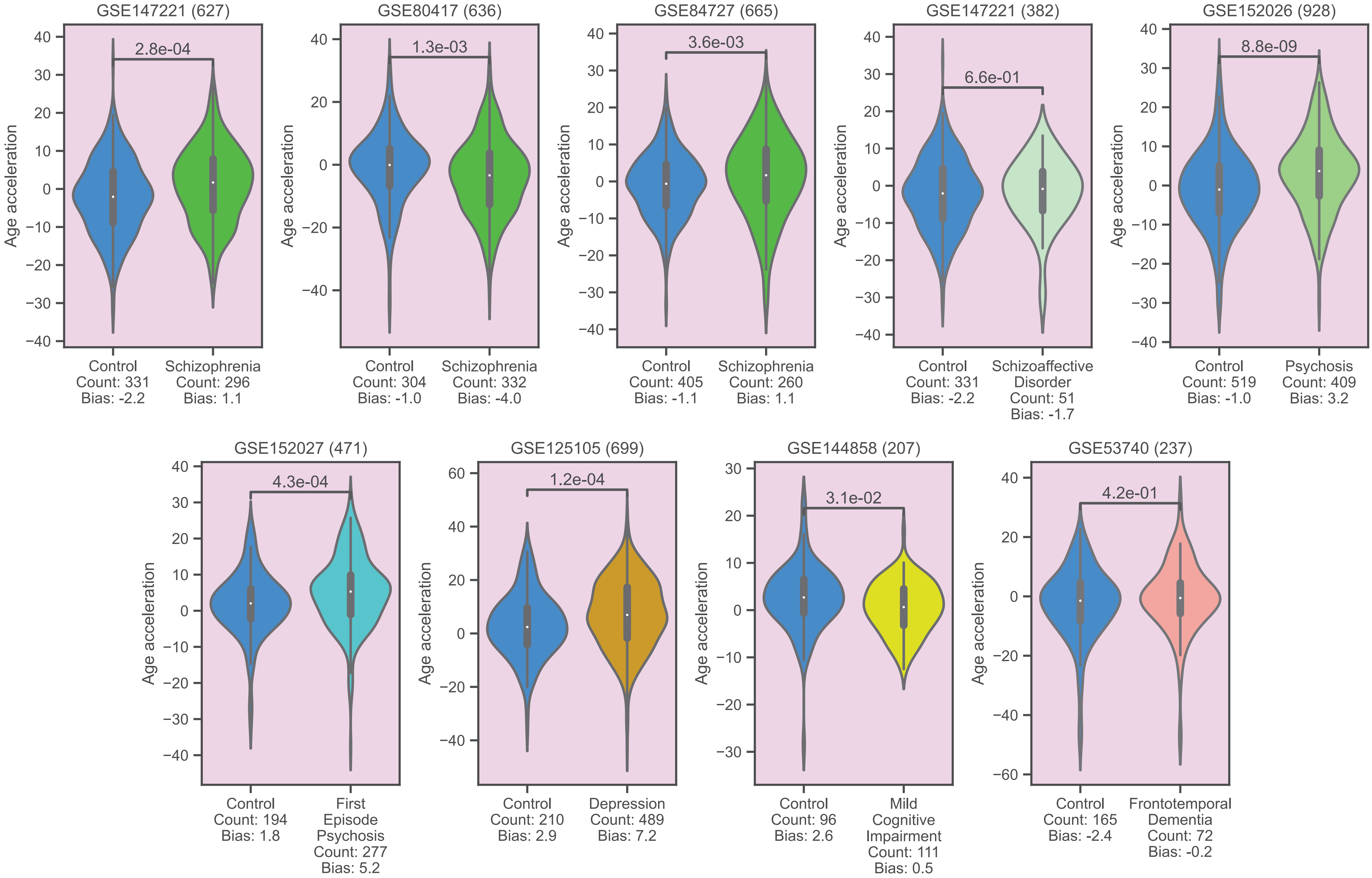

Chapter 8: Diseases of the nervous system

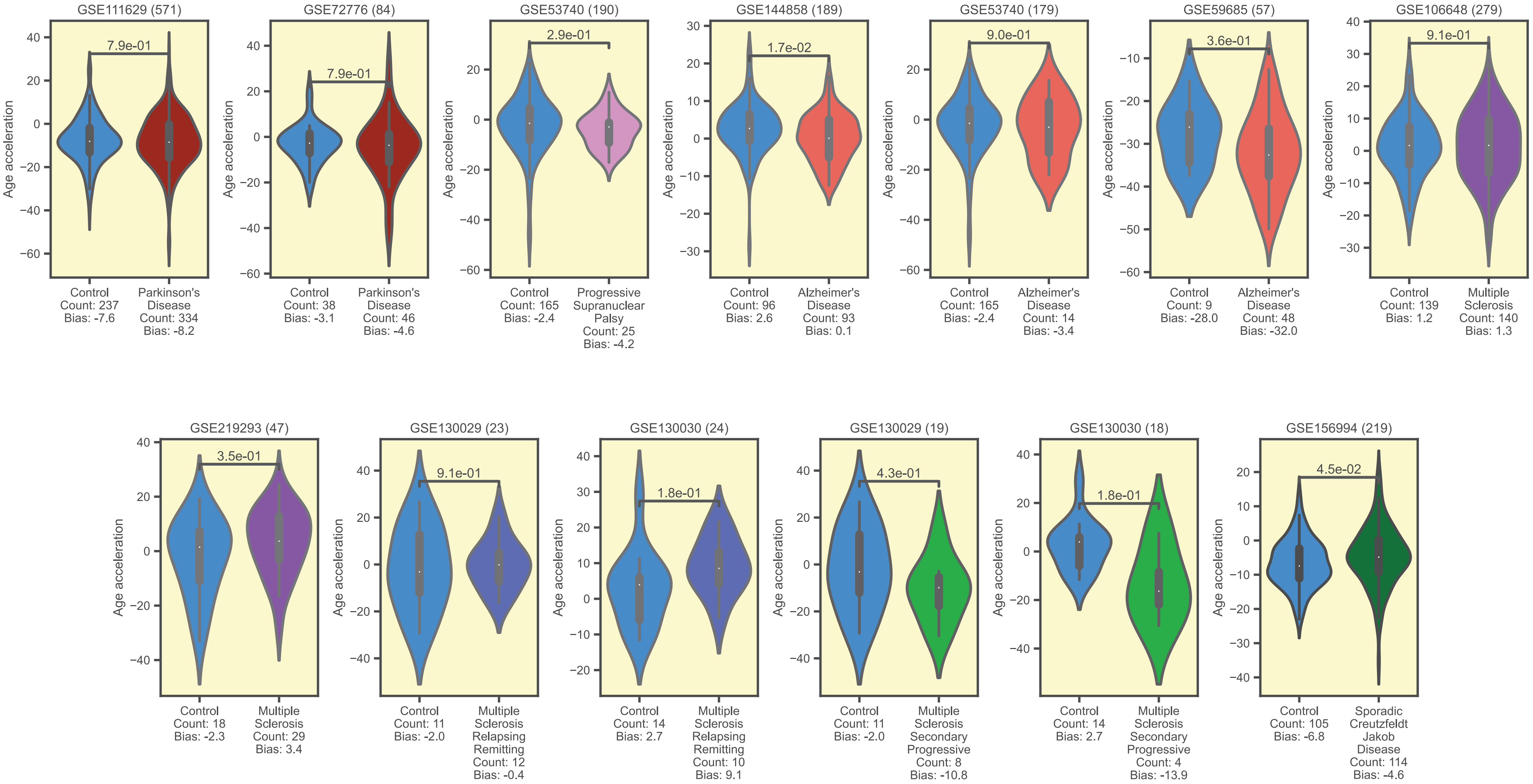

Chapter 11: Diseases of the circulatory system

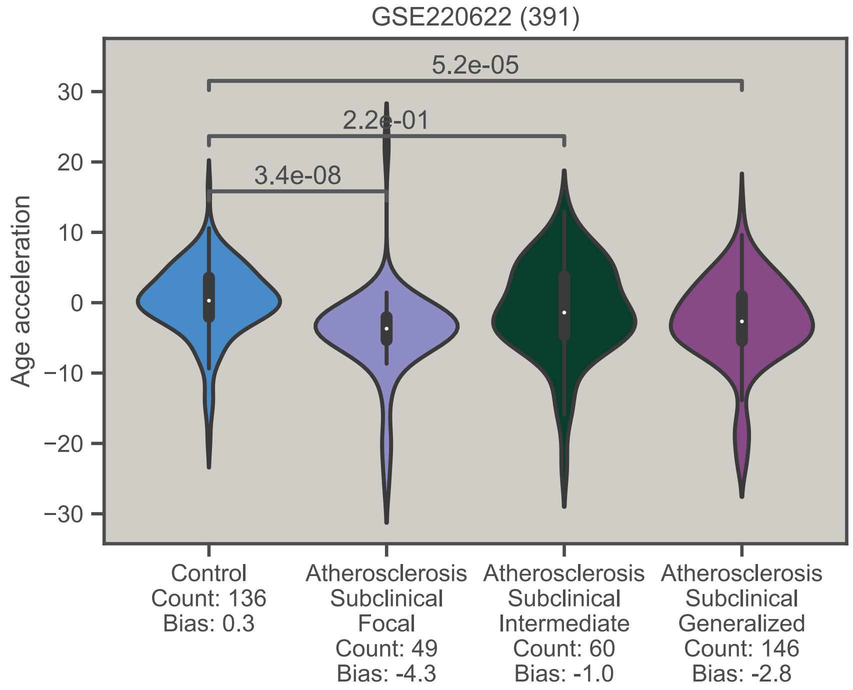

Chapter 12: Diseases of the respiratory system

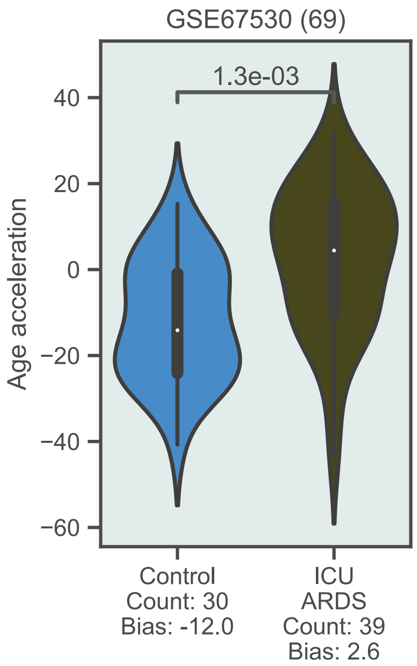

Chapter 13: Diseases of the digestive system

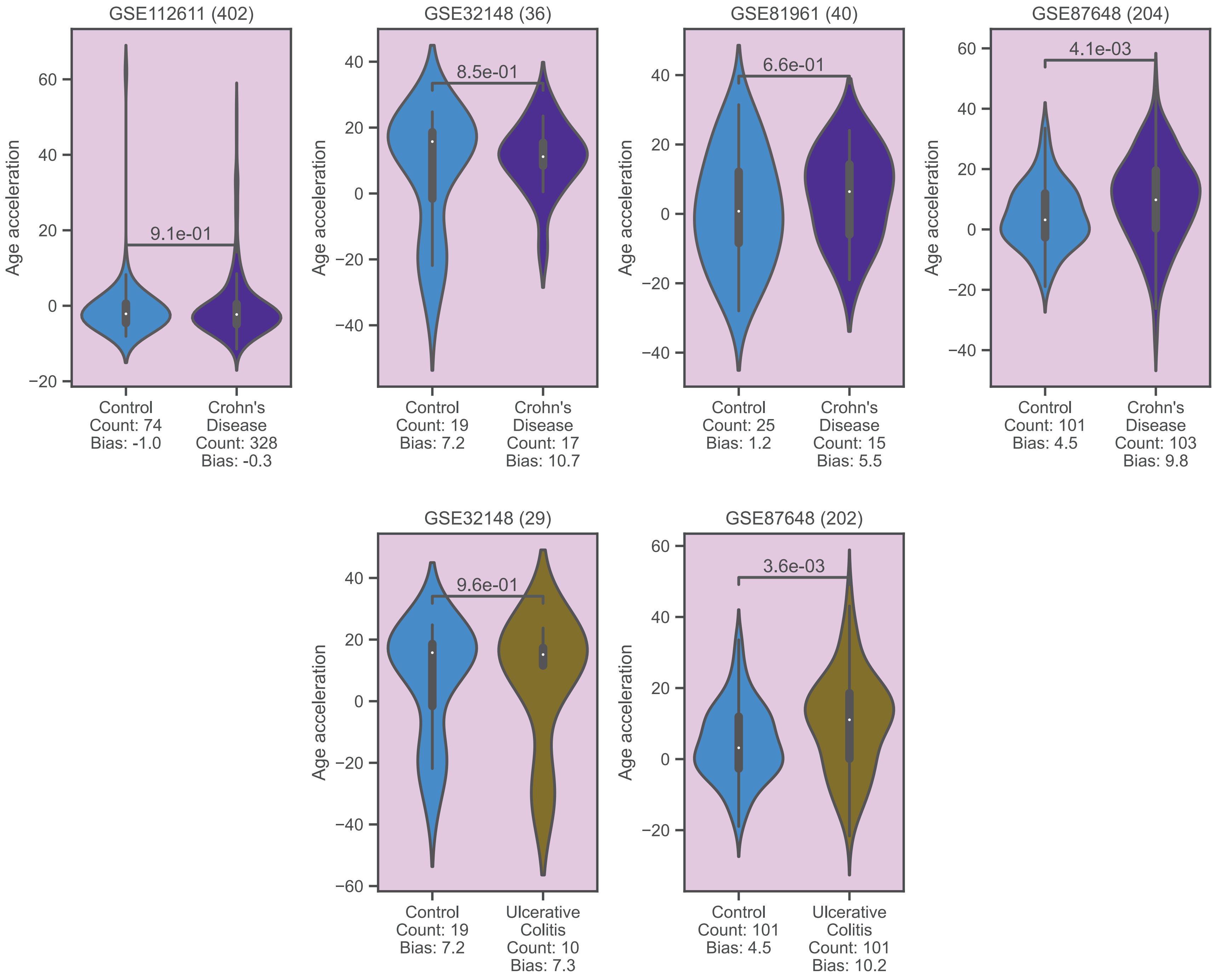

Chapter 15: Diseases of the musculoskeletal system

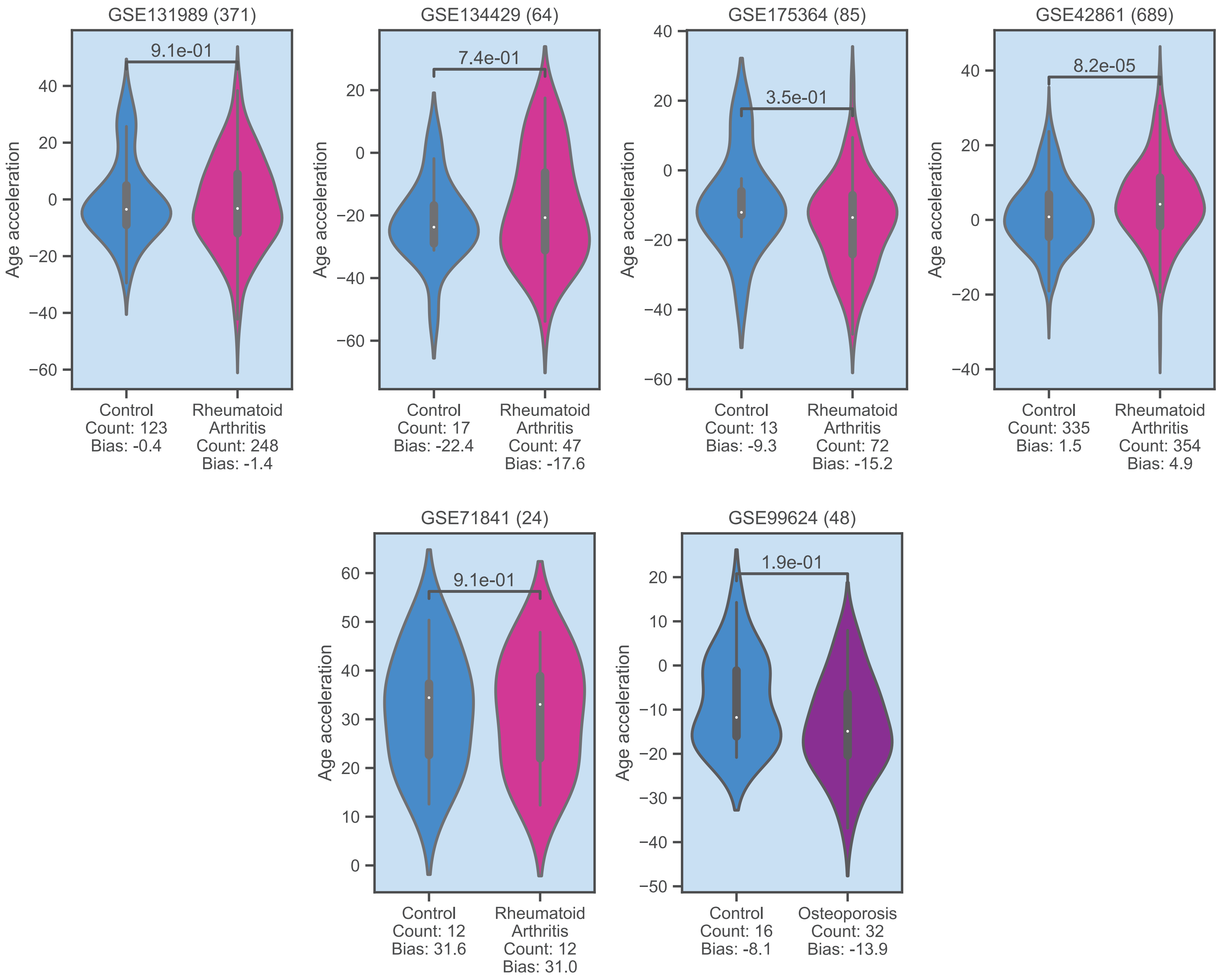

Chapter 16: Diseases of the genitourinary system

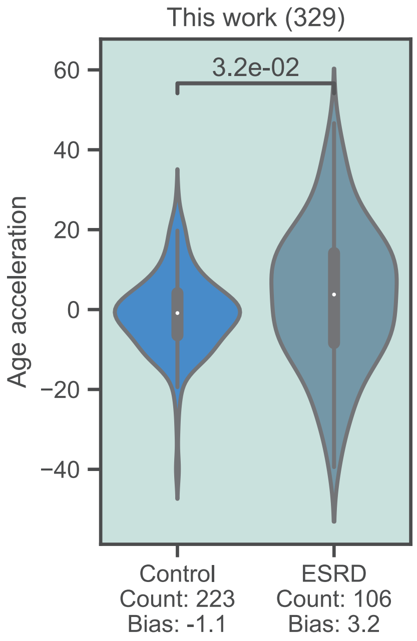

Chapter 20: Developmental anomalies

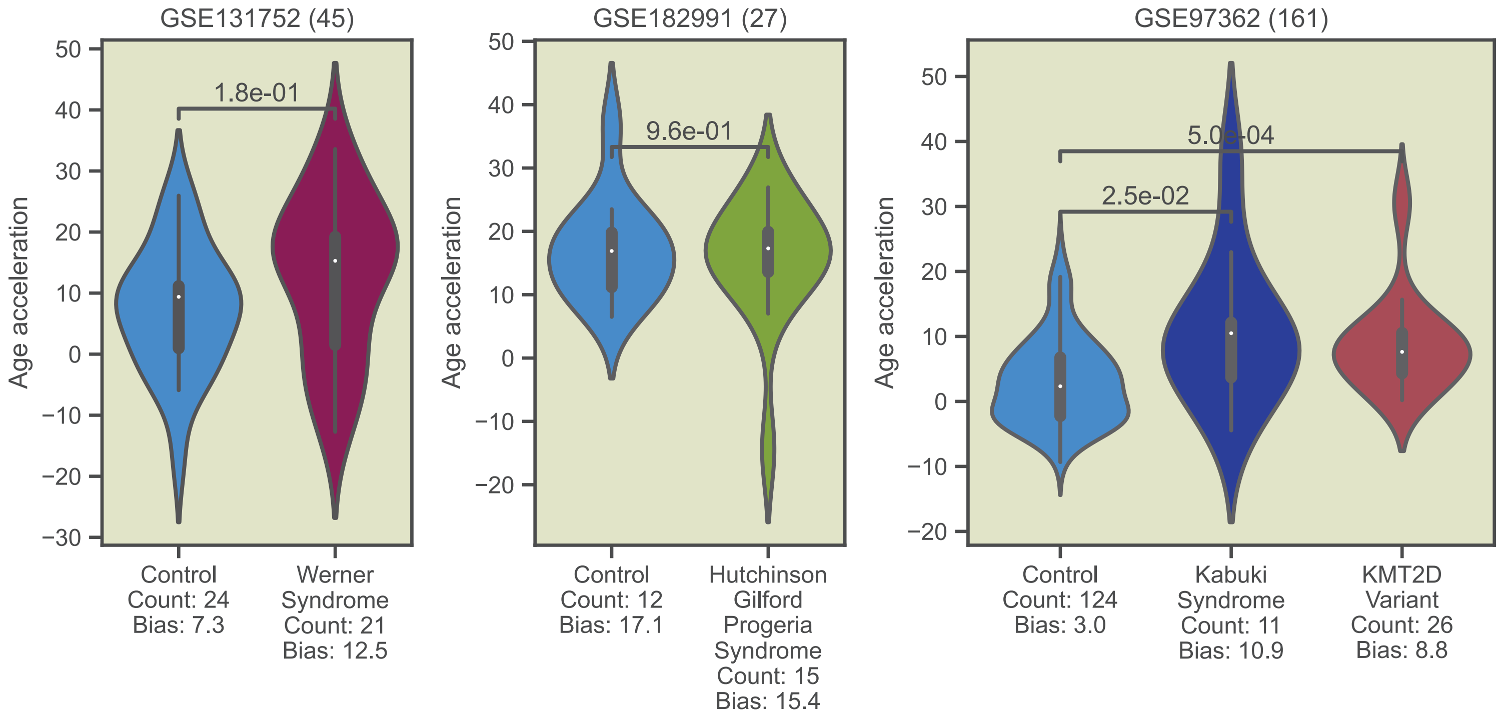

Chapter 25: Codes for special purposes

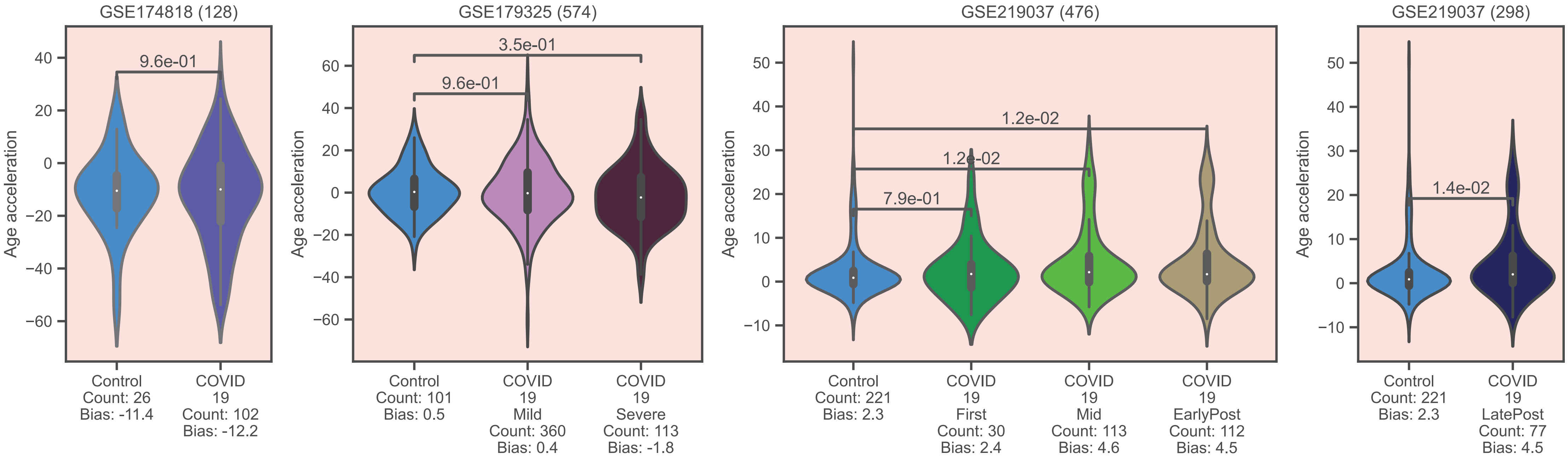

Supplement: Supplementary file 1 [file ijms-26-06284-s001.zip › SupplementaryFigureS3.pdf]
