# Supplementary material for: EpInflammAge: Epigenetic-Inflammatory Clock for Disease-Associated Biological Aging Based on Deep Learning
Source: Int J Mol Sci. 2025 Jun 29;26(13):6284. doi: 10.3390/ijms26136284 (PMC12249966; doi:10.3390/ijms26136284)
Supplement: Supplementary file 1 [file ijms-26-06284-s001.zip › SupplementaryFigureS4.pdf]

					Chapter 1		Chapter 2				Chapter 4		Chapter 5					Chapter 6						Chapter 8							11	12	Chapter 13		Chapter 15		Chapter 16	Chapter 20		Chapter 25	
Clocks	Year	Total Pearson's R	Total MAE	Passed ICD-11 tests	1B10	1C60-1C62	2B90	2B91	2B92	2C60-2C6Z	4A40.0	4A44.2	5A02.0	5A10	5A40	5A61.0	5B81	6A20	6A21	6A23	6A70-6A71	6D71	6D83	8A00.0	8A00.10	8A20	8A40	8A40.0	8A40.2	8E00	BA52, BD40	CB00	DD70	DD71	FA20	FB83.1	GB61.5	LD2B	LD2F.1Y	RA01	RA02
**EpInflammAge**		0.850	7.038	25/76	0/3	5/8	0/1	0/1	0/1	0/1	1/1	0/2	0/1	1/1	0/1	2/2	0/3	2/3	0/1	2/2	1/1	0/1	0/1	0/2	0/1	0/3	0/2	0/2	0/2	1/1	0/3	1/1	1/4	1/2	1/5	0/1	1/1	0/2	2/2	2/6	1/1
**Hannum**	2013	0.896	6.820	14/76	0/3	3/8	1/1	0/1	0/1	0/1	0/1	0/2	0/1	0/1	0/1	0/2	0/3	1/3	0/1	2/2	1/1	0/1	0/1	0/2	1/1	0/3	0/2	0/2	0/2	0/1	0/3	1/1	2/4	1/2	0/5	0/1	1/1	0/2	0/2	0/6	0/1
**Horvath**	2013	0.931	5.425	9/76	0/3	5/8	1/1	0/1	0/1	0/1	0/1	0/2	0/1	0/1	0/1	0/2	0/3	1/3	0/1	1/2	0/1	0/1	0/1	0/2	0/1	0/3	0/2	0/2	0/2	0/1	0/3	0/1	0/4	0/2	0/5	0/1	1/1	0/2	0/2	0/6	0/1
**Lin**	2016	0.899	6.842	10/76	0/3	6/8	0/1	0/1	0/1	0/1	0/1	0/2	0/1	0/1	0/1	0/2	0/3	1/3	0/1	1/2	0/1	0/1	0/1	0/2	1/1	0/3	0/2	0/2	0/2	0/1	0/3	0/1	0/4	0/2	0/5	0/1	0/1	0/2	0/2	0/6	1/1
**epiTOC1**	2016			9/76	0/3	5/8	0/1	0/1	0/1	0/1	0/1	0/2	0/1	0/1	0/1	0/2	1/3	1/3	0/1	1/2	0/1	0/1	0/1	0/2	0/1	0/3	0/2	0/2	0/2	0/1	0/3	0/1	0/4	0/2	0/5	0/1	0/1	0/2	0/2	0/6	1/1
**ZhangMortality**	2017			24/76	0/3	3/8	1/1	0/1	0/1	0/1	0/1	1/2	0/1	0/1	0/1	0/2	0/3	3/3	1/1	0/2	0/1	0/1	1/1	2/2	0/1	1/3	0/2	0/2	0/2	1/1	1/3	1/1	3/4	1/2	3/5	0/1	0/1	0/2	0/2	1/6	0/1
**DNAmPhenoAge**	2018	0.912	8.107	23/76	1/3	5/8	0/1	0/1	0/1	0/1	0/1	0/2	0/1	0/1	0/1	0/2	0/3	2/3	1/1	2/2	1/1	0/1	0/1	0/2	0/1	0/3	0/2	0/2	0/2	1/1	1/3	1/1	2/4	1/2	2/5	0/1	1/1	1/2	0/2	1/6	0/1
**SkinAndBlood**	2018	0.961	3.809	16/76	0/3	5/8	0/1	0/1	0/1	0/1	1/1	0/2	0/1	0/1	0/1	1/2	0/3	1/3	1/1	2/2	0/1	0/1	0/1	0/2	0/1	0/3	0/2	0/2	0/2	0/1	0/3	0/1	0/4	0/2	0/5	0/1	1/1	1/2	0/2	2/6	1/1
**GrimAge**	2019	0.467	16.869	22/76	0/3	3/8	0/1	0/1	0/1	0/1	1/1	0/2	0/1	0/1	0/1	2/2	0/3	2/3	0/1	2/2	1/1	0/1	0/1	0/2	0/1	0/3	1/2	0/2	0/2	1/1	1/3	1/1	2/4	0/2	1/5	0/1	1/1	0/2	1/2	1/6	1/1
**DNAmTL**	2019			17/76	0/3	5/8	1/1	0/1	0/1	0/1	0/1	0/2	0/1	0/1	0/1	0/2	1/3	1/3	0/1	0/2	0/1	0/1	0/1	0/2	0/1	0/3	0/2	0/2	0/2	0/1	3/3	1/1	2/4	0/2	1/5	0/1	0/1	1/2	0/2	1/6	0/1
**ZhangEN**	2019	0.976	6.805	6/76	0/3	3/8	0/1	0/1	0/1	0/1	0/1	0/2	0/1	0/1	0/1	0/2	0/3	1/3	1/1	0/2	0/1	0/1	0/1	0/2	0/1	0/3	0/2	0/2	0/2	0/1	0/3	0/1	1/4	0/2	0/5	0/1	0/1	0/2	0/2	0/6	0/1
**ZhangBLUP**	2019	0.979	2.606	9/76	0/3	4/8	0/1	0/1	0/1	0/1	0/1	0/2	0/1	0/1	0/1	0/2	0/3	1/3	0/1	0/2	1/1	0/1	0/1	0/2	0/1	0/3	0/2	0/2	0/2	0/1	0/3	0/1	1/4	0/2	0/5	0/1	1/1	1/2	0/2	0/6	0/1
**Han**	2020	0.925	5.226	13/76	0/3	6/8	1/1	0/1	0/1	0/1	0/1	0/2	0/1	0/1	0/1	1/2	1/3	1/3	0/1	1/2	0/1	0/1	0/1	0/2	0/1	0/3	0/2	0/2	0/2	0/1	1/3	0/1	0/4	0/2	0/5	0/1	0/1	1/2	0/2	0/6	0/1
**DunedinPACE**	2022			36/76	1/3	5/8	1/1	0/1	0/1	0/1	0/1	1/2	1/1	0/1	0/1	2/2	2/3	3/3	1/1	1/2	0/1	0/1	1/1	0/2	0/1	0/3	1/2	0/2	0/2	1/1	1/3	1/1	4/4	1/2	4/5	0/1	1/1	1/2	0/2	2/6	0/1
**AltumAge**	2022	0.920	5.439	12/76	0/3	4/8	0/1	0/1	0/1	0/1	0/1	0/2	0/1	0/1	0/1	0/2	0/3	0/3	0/1	2/2	0/1	0/1	0/1	0/2	0/1	0/3	0/2	0/2	0/2	1/1	2/3	0/1	0/4	0/2	0/5	0/1	1/1	0/2	0/2	1/6	1/1
**PCHannum**	2022	0.914	8.238	19/76	0/3	6/8	1/1	0/1	0/1	0/1	0/1	0/2	0/1	0/1	0/1	0/2	0/3	2/3	0/1	1/2	1/1	0/1	0/1	0/2	1/1	0/3	0/2	0/2	0/2	1/1	0/3	1/1	2/4	1/2	1/5	0/1	1/1	0/2	0/2	0/6	0/1
**PCHorvath**	2022	0.904	6.827	11/76	0/3	6/8	0/1	0/1	0/1	0/1	0/1	0/2	0/1	0/1	0/1	0/2	0/3	1/3	0/1	1/2	1/1	0/1	0/1	0/2	0/1	0/3	0/2	0/2	0/2	0/1	0/3	0/1	0/4	0/2	0/5	0/1	1/1	0/2	0/2	0/6	1/1
**PCPhenoAge**	2022	0.895	6.478	31/76	2/3	6/8	0/1	0/1	0/1	0/1	0/1	0/2	0/1	0/1	0/1	0/2	0/3	2/3	1/1	2/2	1/1	0/1	1/1	2/2	1/1	0/3	2/2	0/2	0/2	1/1	0/3	1/1	2/4	1/2	1/5	0/1	1/1	1/2	0/2	2/6	1/1
**HRSInCHPhenoAge**	2022	0.913	6.621	34/76	2/3	6/8	1/1	0/1	0/1	0/1	0/1	0/2	0/1	0/1	0/1	0/2	1/3	3/3	1/1	2/2	1/1	0/1	0/1	2/2	1/1	0/3	0/2	0/2	0/2	1/1	1/3	1/1	2/4	1/2	3/5	0/1	1/1	1/2	0/2	2/6	1/1
**PCSkinAndBlood**	2022	0.897	6.627	11/76	0/3	6/8	0/1	0/1	0/1	0/1	0/1	0/2	0/1	0/1	0/1	0/2	0/3	1/3	0/1	1/2	1/1	0/1	0/1	0/2	0/1	0/3	0/2	0/2	0/2	1/1	0/3	0/1	0/4	0/2	0/5	0/1	1/1	0/2	0/2	0/6	0/1
**PCGrimAge**	2022	0.558	24.101	20/76	0/3	3/8	0/1	0/1	0/1	0/1	1/1	0/2	0/1	0/1	0/1	2/2	0/3	2/3	0/1	2/2	1/1	0/1	0/1	0/2	0/1	0/3	0/2	0/2	0/2	1/1	0/3	1/1	2/4	1/2	1/5	0/1	1/1	0/2	1/2	1/6	1/1
**PCDNAmTL**	2022			19/76	0/3	5/8	1/1	0/1	0/1	0/1	0/1	0/2	0/1	0/1	0/1	0/2	1/3	1/3	0/1	0/2	0/1	0/1	0/1	1/2	1/1	0/3	0/2	0/2	0/2	0/1	3/3	0/1	2/4	1/2	1/5	0/1	0/1	1/2	0/2	1/6	0/1
**GrimAge2**	2022	0.529	19.419	27/76	0/3	3/8	0/1	0/1	0/1	0/1	1/1	0/2	0/1	0/1	0/1	2/2	0/3	2/3	0/1	2/2	1/1	0/1	0/1	0/2	0/1	0/3	1/2	0/2	0/2	1/1	1/3	1/1	3/4	1/2	1/5	0/1	1/1	0/2	2/2	3/6	1/1
**DNAmFitAge**	2023	0.888	14.558	12/76	0/3	6/8	0/1	0/1	0/1	0/1	1/1	0/2	0/1	0/1	0/1	0/2	0/3	1/3	0/1	2/2	1/1	0/1	0/1	0/2	0/1	0/3	0/2	0/2	0/2	1/1	0/3	0/1	0/4	0/2	0/5	0/1	0/1	0/2	0/2	0/6	0/1
**ENCen40**	2023	0.959	5.345	14/76	0/3	6/8	0/1	0/1	0/1	0/1	0/1	0/2	0/1	0/1	0/1	0/2	0/3	1/3	0/1	2/2	0/1	0/1	0/1	0/2	1/1	0/3	0/2	0/2	0/2	0/1	0/3	0/1	1/4	0/2	0/5	0/1	0/1	1/2	0/2	1/6	1/1
**YingAdaptAge**	2024	0.559	17.244	8/76	0/3	3/8	0/1	0/1	0/1	1/1	0/1	0/2	0/1	0/1	0/1	0/2	0/3	1/3	0/1	2/2	0/1	0/1	0/1	0/2	0/1	0/3	0/2	0/2	0/2	0/1	0/3	0/1	1/4	0/2	0/5	0/1	0/1	0/2	0/2	0/6	0/1
**YingCausAge**	2024	0.589	23.329	13/76	0/3	6/8	0/1	0/1	0/1	0/1	0/1	0/2	0/1	0/1	0/1	0/2	0/3	2/3	0/1	1/2	0/1	0/1	0/1	0/2	1/1	0/3	0/2	0/2	0/2	0/1	0/3	0/1	1/4	0/2	0/5	0/1	0/1	1/2	0/2	0/6	1/1
**YingDamAge**	2024	0.467	18.330	11/76	0/3	1/8	0/1	0/1	0/1	0/1	0/1	0/2	0/1	0/1	0/1	0/2	0/3	1/3	0/1	2/2	0/1	0/1	0/1	0/2	0/1	0/3	0/2	0/2	0/2	0/1	0/3	1/1	1/4	0/2	0/5	0/1	1/1	0/2	0/2	3/6	1/1
**StocH**	2024	0.702	10.455	6/76	0/3	4/8	0/1	0/1	0/1	0/1	0/1	0/2	0/1	0/1	0/1	0/2	0/3	0/3	0/1	2/2	0/1	0/1	0/1	0/2	0/1	0/3	0/2	0/2	0/2	0/1	0/3	0/1	0/4	0/2	0/5	0/1	0/1	0/2	0/2	0/6	0/1
**StocP**	2024	0.755	10.122	7/76	0/3	4/8	0/1	0/1	0/1	0/1	0/1	0/2	0/1	0/1	0/1	0/2	0/3	0/3	0/1	1/2	0/1	0/1	0/1	0/2	0/1	0/3	0/2	0/2	0/2	0/1	0/3	0/1	0/4	0/2	0/5	0/1	1/1	0/2	1/2	0/6	0/1
**StocZ**	2024	0.942	11.687	7/76	0/3	5/8	0/1	0/1	0/1	0/1	0/1	0/2	0/1	0/1	0/1	0/2	1/3	0/3	0/1	0/2	0/1	0/1	0/1	1/2	0/1	0/3	0/2	0/2	0/2	0/1	0/3	0/1	0/4	0/2	0/5	0/1	0/1	0/2	0/2	0/6	0/1
**stemTOC**	2024			6/76	0/3	5/8	0/1	0/1	0/1	0/1	0/1	0/2	0/1	0/1	0/1	0/2	0/3	0/3	0/1	1/2	0/1	0/1	0/1	0/2	0/1	0/3	0/2	0/2	0/2	0/1	0/3	0/1	0/4	0/2	0/5	0/1	0/1	0/2	0/2	0/6	0/1
**RetroelementAgeV1**	2024	0.780	27.900	11/76	0/3	6/8	0/1	0/1	0/1	0/1	1/1	0/2	0/1	0/1	0/1	0/2	0/3	0/3	0/1	2/2	1/1	0/1	0/1	0/2	0/1	0/3	0/2	0/2	0/2	0/1	0/3	0/1	0/4	0/2	0/5	0/1	0/1	1/2	0/2	0/6	0/1
**RetroelementAgeV2**	2024	0.806	12.017	9/76	0/3	2/8	0/1	0/1	0/1	0/1	1/1	0/2	0/1	0/1	0/1	1/2	0/3	0/3	0/1	2/2	1/1	0/1	0/1	0/2	0/1	0/3	0/2	0/2	0/2	0/1	0/3	0/1	0/4	0/2	0/5	0/1	0/1	1/2	1/2	0/6	0/1
**IntrinClock**	2024	0.960	3.719	7/76	0/3	2/8	0/1	0/1	0/1	0/1	0/1	0/2	0/1	0/1	0/1	0/2	0/3	1/3	0/1	0/2	0/1	0/1	0/1	0/2	0/1	0/3	0/2	0/2	0/2	0/1	0/3	0/1	0/4	0/2	0/5	0/1	1/1	1/2	0/2	1/6	1/1
