# Supplementary material for: EpInflammAge: Epigenetic-Inflammatory Clock for Disease-Associated Biological Aging Based on Deep Learning
Source: Int J Mol Sci. 2025 Jun 29;26(13):6284. doi: 10.3390/ijms26136284 (PMC12249966; doi:10.3390/ijms26136284)
Supplement: Supplementary file 1 [file ijms-26-06284-s001.zip › SupplementaryFigureS6.pdf]

Inflammatory marker	Passed ICD-11 tests	Chapter 1		Chapter 2				Chapter 4		Chapter 5					Chapter 6						Chapter 8							11	12	Chapter 13		Chapter 15		Chapter 16	Chapter 20		Chapter 25	
		1B10	1C60-1C62	2B90	2B91	2B92	2C60-2C6Z	4A40.0	4A44.2	5A02.0	5A10	5A40	5A61.0	5B81	6A20	6A21	6A23	6A70-6A71	6D71	6D83	8A00.0	8A00.10	8A20	8A40	8A40.0	8A40.2	8E00	BA52, BD40	CB00	DD70	DD71	FA20	FB83.1	GB61.5	LD2B	LD2F.1Y	RA01	RA02
CXCL9	25/76	0/3	2/8	0/1	0/1	0/1	1/1	0/1	0/2	1/1	0/1	0/1	0/2	2/3	3/3	1/1	1/2	0/1	0/1	0/1	1/2	0/1	0/3	1/2	0/2	0/2	0/1	1/3	1/1	2/4	1/2	1/5	0/1	1/1	1/2	0/2	3/6	1/1
CCL11	2/76	0/3	0/8	0/1	0/1	0/1	0/1	0/1	0/2	0/1	0/1	1/1	0/2	0/3	0/3	0/1	0/2	0/1	0/1	0/1	0/2	0/1	0/3	0/2	0/2	0/2	0/1	0/3	0/1	0/4	0/2	0/5	0/1	1/1	0/2	0/2	0/6	0/1
IL27	14/76	0/3	2/8	0/1	0/1	0/1	1/1	0/1	0/2	0/1	0/1	0/1	0/2	2/3	1/3	0/1	0/2	0/1	0/1	0/1	2/2	0/1	0/3	0/2	0/2	0/2	0/1	0/3	0/1	1/4	1/2	1/5	0/1	1/1	0/2	1/2	1/6	0/1
IL5	2/76	0/3	0/8	0/1	0/1	0/1	0/1	0/1	0/2	0/1	0/1	0/1	0/2	0/3	0/3	0/1	0/2	0/1	0/1	0/1	0/2	0/1	0/3	0/2	0/2	0/2	0/1	0/3	0/1	0/4	0/2	1/5	0/1	0/1	0/2	0/2	1/6	0/1
CSF1	11/76	0/3	1/8	0/1	0/1	0/1	0/1	1/1	0/2	1/1	0/1	0/1	0/2	0/3	1/3	0/1	1/2	0/1	0/1	0/1	0/2	0/1	0/3	1/2	0/2	0/2	0/1	0/3	1/1	0/4	0/2	0/5	0/1	1/1	0/2	0/2	2/6	1/1
CCL2	15/76	1/3	2/8	0/1	0/1	0/1	0/1	0/1	0/2	0/1	0/1	0/1	0/2	2/3	3/3	0/1	0/2	0/1	0/1	0/1	0/2	0/1	0/3	0/2	0/2	0/2	0/1	0/3	1/1	0/4	0/2	1/5	0/1	1/1	0/2	1/2	2/6	1/1
IL1B	11/76	0/3	4/8	0/1	0/1	0/1	1/1	0/1	0/2	0/1	0/1	0/1	0/2	1/3	1/3	0/1	1/2	0/1	0/1	0/1	0/2	0/1	0/3	0/2	0/2	0/2	0/1	0/3	0/1	0/4	0/2	0/5	0/1	1/1	0/2	0/2	2/6	0/1
IL6	24/76	0/3	3/8	1/1	0/1	0/1	0/1	0/1	0/2	0/1	0/1	0/1	2/2	1/3	3/3	1/1	1/2	0/1	0/1	0/1	1/2	1/1	0/3	0/2	0/2	0/2	1/1	2/3	1/1	1/4	1/2	2/5	0/1	1/1	0/2	0/2	1/6	0/1
GCSF	6/76	0/3	1/8	0/1	0/1	0/1	1/1	0/1	0/2	0/1	0/1	0/1	0/2	0/3	0/3	0/1	0/2	0/1	0/1	0/1	1/2	0/1	0/3	0/2	0/2	0/2	0/1	0/3	0/1	0/4	0/2	1/5	0/1	1/1	0/2	0/2	1/6	0/1
CXCL10	17/76	0/3	4/8	0/1	0/1	0/1	1/1	0/1	0/2	0/1	0/1	1/1	1/2	0/3	3/3	0/1	0/2	0/1	0/1	0/1	1/2	1/1	0/3	0/2	0/2	0/2	0/1	0/3	1/1	1/4	1/2	1/5	0/1	0/1	0/2	0/2	1/6	0/1
VEGFA	7/76	0/3	3/8	0/1	0/1	0/1	0/1	0/1	0/2	0/1	0/1	0/1	0/2	0/3	0/3	0/1	0/2	0/1	0/1	0/1	0/2	0/1	0/3	0/2	0/2	0/2	1/1	0/3	0/1	0/4	0/2	0/5	0/1	0/1	0/2	0/2	2/6	1/1
TNF	8/76	0/3	2/8	0/1	0/1	0/1	0/1	0/1	0/2	0/1	0/1	0/1	0/2	0/3	0/3	0/1	0/2	0/1	0/1	0/1	0/2	0/1	0/3	0/2	0/2	0/2	0/1	0/3	0/1	2/4	0/2	1/5	0/1	0/1	0/2	0/2	2/6	1/1
PDGFB	4/76	0/3	1/8	1/1	0/1	0/1	0/1	0/1	0/2	0/1	0/1	0/1	0/2	0/3	0/3	0/1	0/2	0/1	0/1	0/1	0/2	0/1	0/3	0/2	0/2	0/2	0/1	0/3	0/1	0/4	0/2	0/5	0/1	1/1	0/2	0/2	1/6	0/1
IL8	9/76	0/3	2/8	0/1	0/1	0/1	1/1	0/1	0/2	0/1	0/1	0/1	1/2	0/3	1/3	0/1	0/2	0/1	0/1	0/1	0/2	0/1	0/3	0/2	0/2	0/2	0/1	0/3	1/1	0/4	0/2	0/5	0/1	0/1	0/2	0/2	2/6	1/1
PDGFA	0/76	0/3	0/8	0/1	0/1	0/1	0/1	0/1	0/2	0/1	0/1	0/1	0/2	0/3	0/3	0/1	0/2	0/1	0/1	0/1	0/2	0/1	0/3	0/2	0/2	0/2	0/1	0/3	0/1	0/4	0/2	0/5	0/1	0/1	0/2	0/2	0/6	0/1
IL12Bp40	5/76	0/3	1/8	0/1	0/1	0/1	0/1	1/1	0/2	0/1	0/1	0/1	0/2	0/3	0/3	0/1	1/2	0/1	0/1	0/1	1/2	0/1	0/3	0/2	0/2	0/2	0/1	0/3	0/1	0/4	0/2	0/5	0/1	1/1	0/2	0/2	0/6	0/1
IL15	17/76	0/3	5/8	0/1	0/1	0/1	0/1	0/1	1/2	1/1	0/1	0/1	1/2	0/3	1/3	0/1	0/2	1/1	0/1	0/1	0/2	0/1	0/3	0/2	0/2	0/2	1/1	0/3	0/1	2/4	0/2	1/5	0/1	1/1	0/2	0/2	1/6	1/1
CXCL1	7/76	0/3	1/8	0/1	0/1	0/1	1/1	0/1	0/2	0/1	0/1	0/1	0/2	0/3	0/3	0/1	1/2	0/1	0/1	0/1	0/2	0/1	0/3	0/2	0/2	0/2	0/1	0/3	0/1	0/4	0/2	1/5	0/1	0/1	0/2	0/2	2/6	1/1
CCL4	3/76	0/3	1/8	0/1	0/1	0/1	0/1	0/1	0/2	0/1	0/1	0/1	0/2	0/3	0/3	0/1	0/2	0/1	0/1	0/1	0/2	0/1	1/3	0/2	0/2	0/2	0/1	0/3	0/1	0/4	0/2	1/5	0/1	0/1	0/2	0/2	0/6	0/1
IFNA2	5/76	0/3	0/8	0/1	0/1	0/1	1/1	0/1	0/2	0/1	0/1	0/1	0/2	2/3	1/3	0/1	0/2	0/1	0/1	0/1	0/2	0/1	0/3	0/2	0/2	0/2	0/1	0/3	0/1	0/4	0/2	1/5	0/1	0/1	0/2	0/2	0/6	0/1
IL13	7/76	0/3	0/8	1/1	0/1	0/1	1/1	0/1	0/2	0/1	0/1	0/1	1/2	0/3	0/3	0/1	0/2	0/1	0/1	0/1	0/2	0/1	0/3	0/2	0/2	1/2	0/1	0/3	0/1	0/4	0/2	1/5	0/1	0/1	0/2	0/2	2/6	0/1
FLT3L	11/76	0/3	5/8	0/1	0/1	0/1	0/1	0/1	0/2	0/1	0/1	0/1	0/2	0/3	0/3	0/1	1/2	0/1	0/1	0/1	0/2	1/1	0/3	1/2	0/2	0/2	0/1	0/3	0/1	0/4	1/2	0/5	0/1	1/1	0/2	1/2	0/6	0/1
CD40LG	4/76	0/3	0/8	0/1	0/1	0/1	0/1	1/1	0/2	0/1	0/1	0/1	0/2	0/3	1/3	0/1	0/2	0/1	0/1	0/1	0/2	0/1	0/3	0/2	0/2	1/2	0/1	0/3	0/1	0/4	0/2	0/5	0/1	1/1	0/2	0/2	0/6	0/1
CCL22	11/76	0/3	0/8	0/1	0/1	0/1	1/1	0/1	1/2	0/1	0/1	0/1	1/2	0/3	1/3	0/1	0/2	0/1	0/1	0/1	0/2	1/1	0/3	0/2	0/2	0/2	1/1	0/3	0/1	0/4	0/2	1/5	0/1	1/1	0/2	0/2	2/6	0/1
