# Supplementary figures and images for: EpInflammAge: Epigenetic-Inflammatory Clock for Disease-Associated Biological Aging Based on Deep Learning
Source: Int J Mol Sci. 2025 Jun 29;26(13):6284. doi: 10.3390/ijms26136284 (PMC12249966; doi:10.3390/ijms26136284)

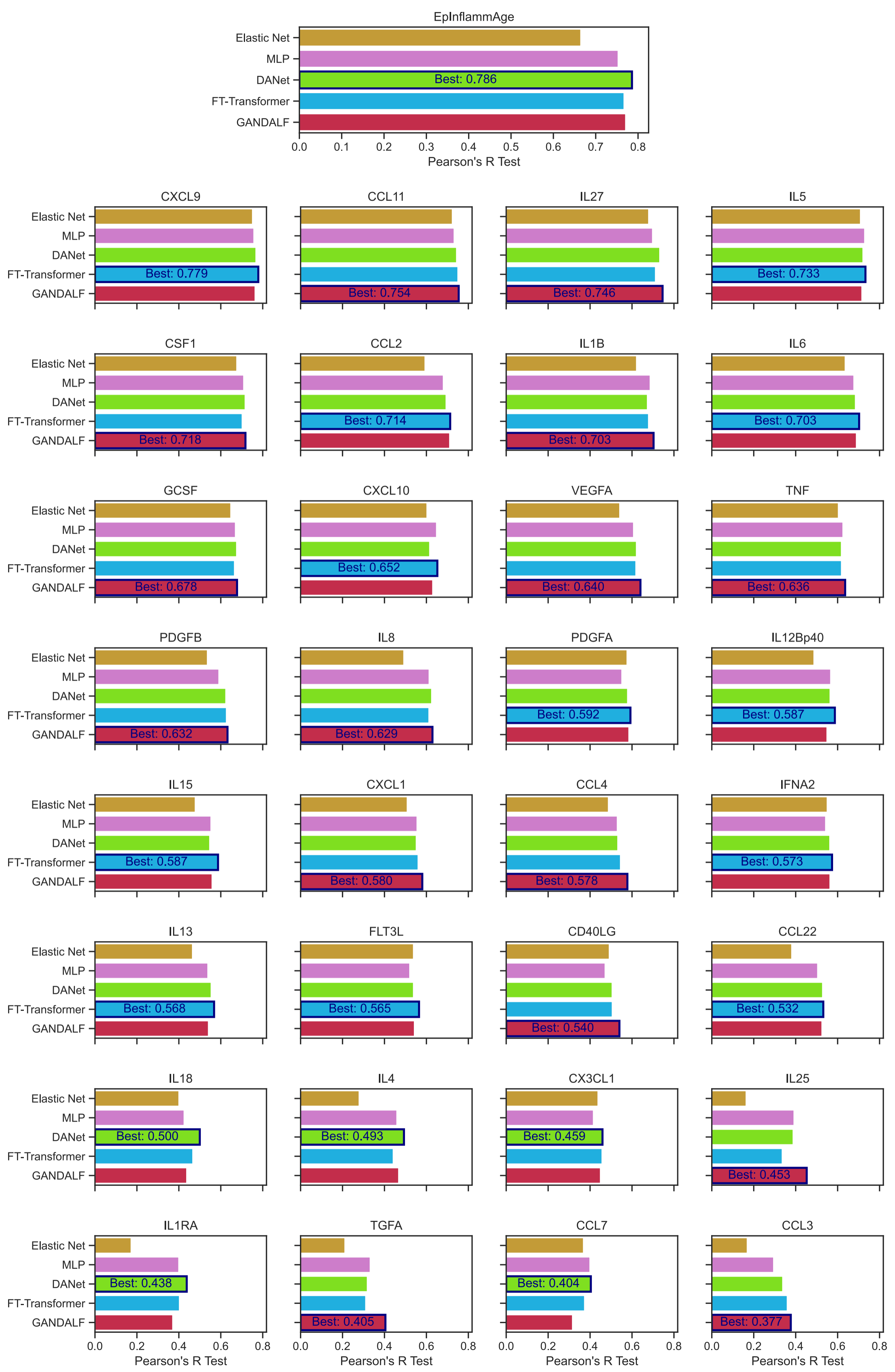

Supplement: Supplementary file 1 [file ijms-26-06284-s001.zip › SupplementaryFigureS1.pdf]

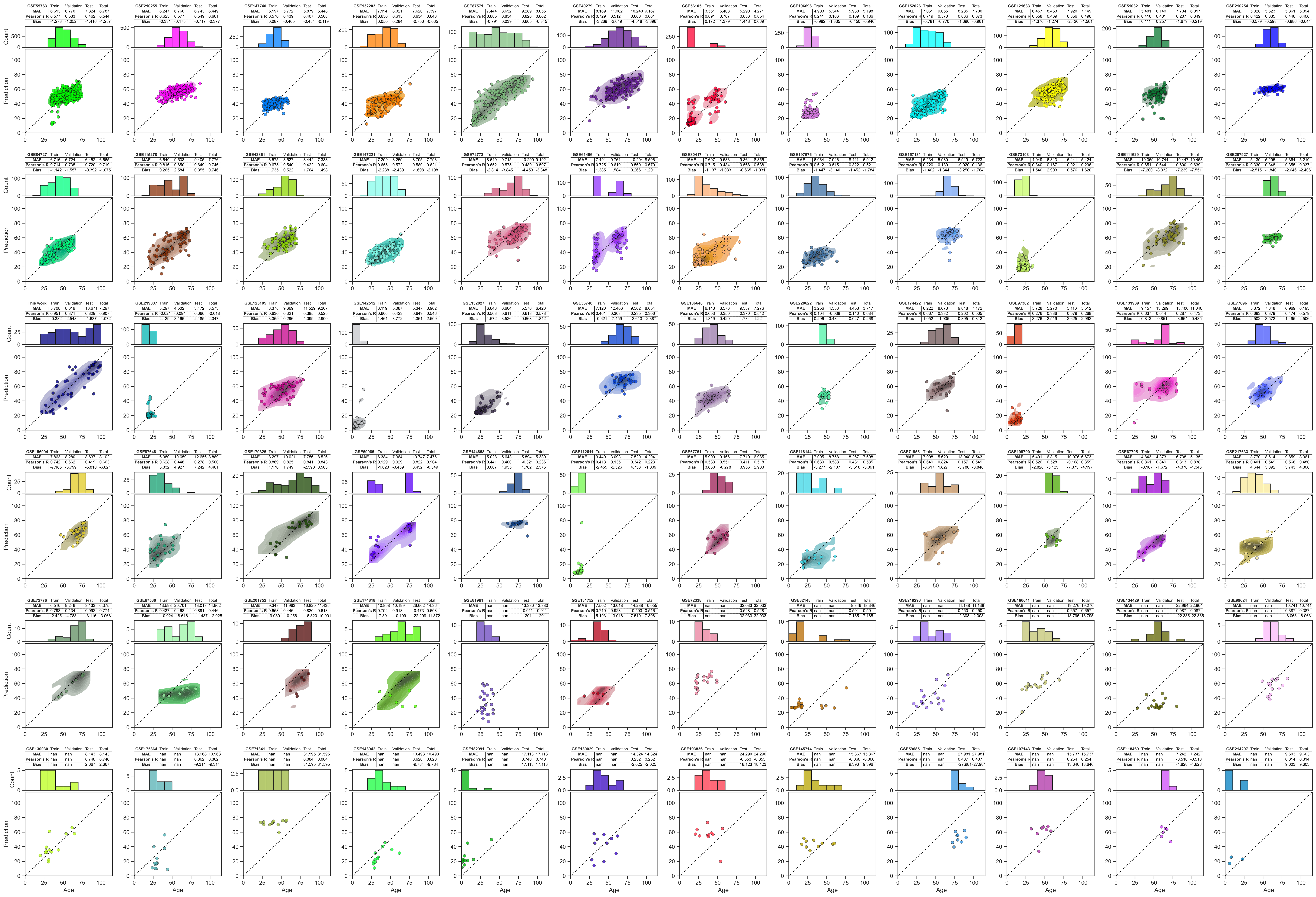

Supplement: Supplementary file 1 [file ijms-26-06284-s001.zip › SupplementaryFigureS2.pdf]

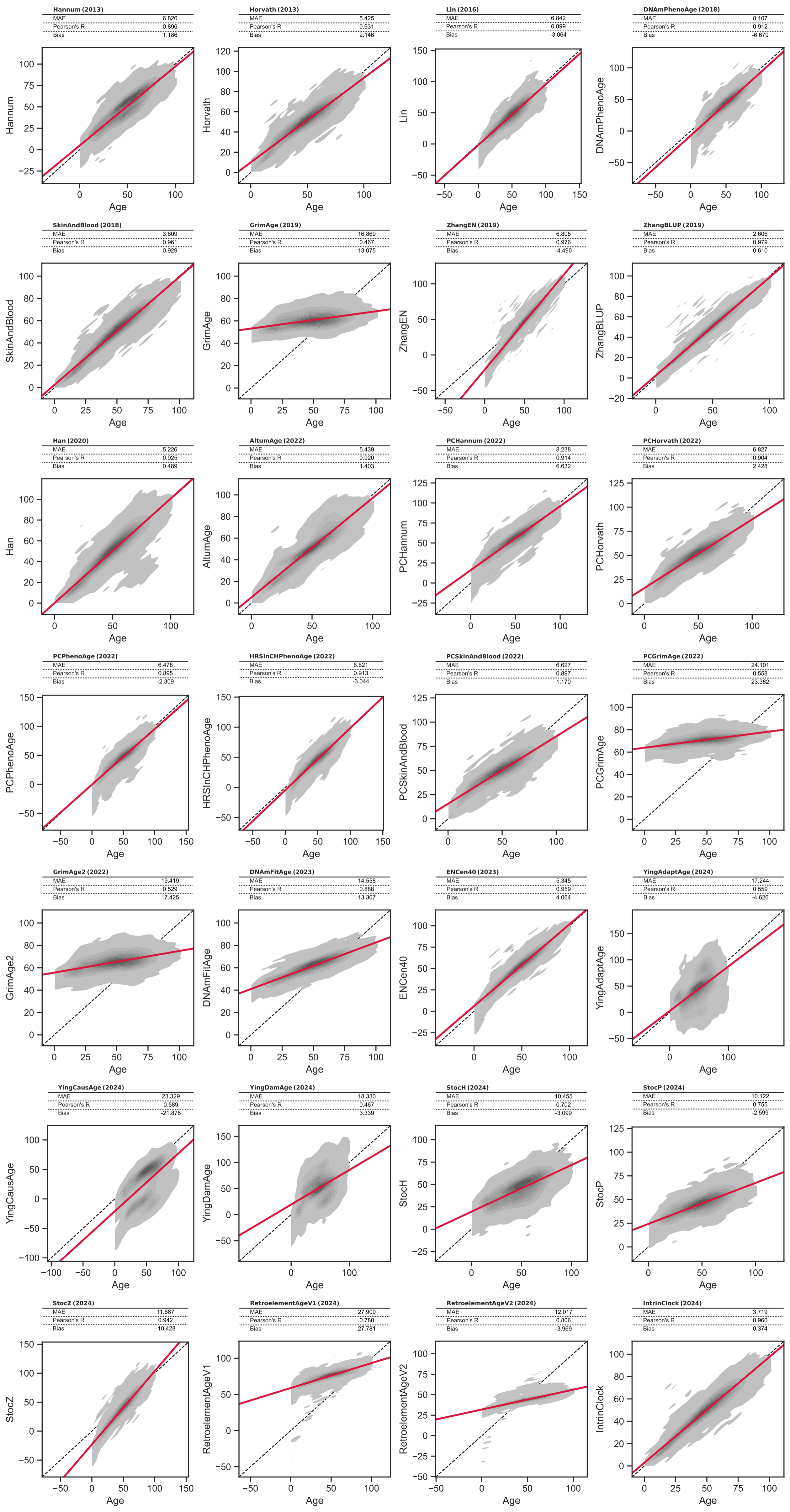

Supplement: Supplementary file 1 [file ijms-26-06284-s001.zip › SupplementaryFigureS5.pdf]
